# Stability and decay of subradiant patterns in a quantum gas with photon-mediated interactions

**DOI:** 10.1126/sciadv.adw0299

**Published:** 2025-07-16

**Authors:** Alexander Baumgärtner, Simon Hertlein, Tom Schmit, Davide Dreon, Carlos Máximo, Xiangliang Li, Giovanna Morigi, Tobias Donner

**Affiliations:** ^1^ Institute for Quantum Electronics, Eidgenössische Technische Hochschule Zürich, Otto-Stern-Weg 1, CH-8093 Zurich, Switzerland.; ^2^ Theoretical Physics, Saarland University, Campus E2.6, D-66123 Saarbrücken, Germany.

## Abstract

Metastability and its relaxation mechanisms challenge our understanding of the stability of quantum many-body systems, revealing a gap between the microscopic dynamics of the individual components and the effective descriptions used for macroscopic observables. We observe excited self-ordered subradiant patterns in a quantum gas coupled to two optical cavities and report lifetimes far beyond the system’s typical timescales. These patterns eventually decay through an abrupt transition reordering the atoms into a superradiant phase. Ab initio theory fully captures this macroscopic behavior, revealing that the subradiant patterns are stabilized by photon-mediated long-range interactions, thereby manifesting universal features of metastability characteristic of long-range interacting systems, as in astrophysics and plasma physics. Our work sheds light on the microscopic mechanisms stabilizing quantum states of matter and highlights the potential of photon-mediated forces for engineering correlations in many-body quantum systems.

## Teaser

Long-lived subradiant patterns in a quantum gas reveal metastability driven by photon-mediated interactions.

## INTRODUCTION

Collective phenomena are ubiquitous in nature, influencing systems as diverse as material properties, economic markets, and galaxy formation. They typically emerge far from equilibrium, where metastability and relaxation dynamics play a crucial role in state transitions. Manifestations are the emergence of organized patterns, critical dynamics, and temporal phases in complex systems. Their understanding and control motivates the need to identify universal principles that underlie these behaviors. In the atomic realm, subradiance and superradiance offer notable examples of how collective effects manifest themselves in light-scattering ensembles, demonstrating the impact of synchronized and desynchronized interactions at microscopic scales (*1*–*4*). Subradiance occurs when emitters, in an antisymmetric superposition, oscillate out of phase and decouple from their electromagnetic environment, suppressing emission rates (*5*–*12*). Conversely, superradiance arises from symmetric superpositions, enhancing emission through constructive interference (*2*, *13*).

Enhancement or suppression of light scattering is controlled by the relative phase among the emitters, which is typically determined by their spatial arrangement (*14*, *15*). In atomic and molecular gases, superradiant states can self-enhance as the mechanical forces of the scattered light pulls emitters into phase-coherent patterns (*16*). In optical cavities, light scattering creates effective long-range forces that even can stabilize ordered configurations of ultra-cold atomic gases (*17*, *18*). One prominent example is the transition from a disordered superfluid to a self-ordered supersolid in a quantum gas coupled to a mode of the electromagnetic field of a high-finesse cavity, which is paradigmatic of the Dicke quantum phase transition (*19*–*23*). However, such self-organization mechanisms are generally inaccessible for subradiant ensembles, as scattered light—and consequently, optical forces—vanishes. For this reason, so far, only transient subradiant states have been observed in itinerant systems (*24*–*29*), while stable subradiant patterns have been achieved by pinning the atoms using external potentials (*15*, *30*, *31*).

**Fig. 1.**
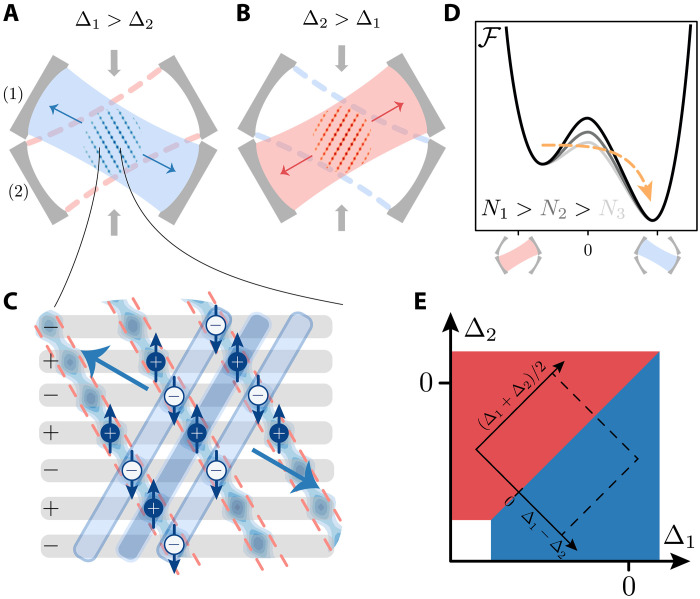
Subradiant state of a BEC in two crossed cavities. (**A** and **B**) Self-ordered BEC (blue, red structures) simultaneously coupled to two optical cavities (1 and 2) and transverse pump lattice (gray arrows). Depending on the relative detunings $\Delta_{1,2}$ between pump frequency and cavity resonances, the equilibrium structure scatters light either into cavity 1 or 2. (**C**) Microscopic illustration of the collective scattering. The gray lines indicate the standing wave pump field, and its time phase is indicated by the ( ± ) signs. The atoms (density pattern/spheres) oscillate with the driving field and order in stripes that maximize emission into cavity 1, where all atoms within a plane (blue boxes) emit with the same phase. At the same time, they suppress scattering into cavity 2, where neighboring atoms emit in opposition of phase (red dashed planes). (**D**) Free energy landscape illustrating the self-organized potential confining the subradiant phase. The barrier separates the subradiant from the superradiant phase, and its height is determined by the atom number (see gray shading of lines). Atom losses lower the barrier and induce an abrupt transition into the superradiant order. (**E**) Illustration of the stationary phase diagram (blue and red regions correspond to ordering in cavity 1 or 2, respectively) as a function of detunings $\Delta_{1,2}$ and for a fixed pump amplitude. The rotated axis and the dashed rectangle sketch the coordinate system used in the subsequent figures. The points where the bare cavity detunings vanish, $\Delta_{1,2}=0$ , lie within the self-ordered phases since the dispersive coupling with the medium shifts the cavity resonances by several megahertz.

In this work, we report the measurement of a self-stabilized subradiant many-body phase. The phase is observed in a quantum gas interacting with two cavity fields, imposing competing order, as illustrated in Fig. 1 (A and B). The subradiant pattern is stabilized by the mechanical forces due to the coupling with a secondary cavity field, which pins the atoms in a pattern inhibiting emission into a primary cavity, as shown in Fig. 1C. The atoms remain locked into the subradiant pattern for exceptionally long times, even when the state is energetically unfavorable. The subradiant state eventually decays by means of a violent relaxation, at which point the gas abruptly reorganizes into a superradiant, stationary pattern. We find that atomic losses are solely responsible for the finite lifetime of the subradiant states, triggering a nonequilibrium, driven-dissipative phase transition from subradiance to superradiance. These characteristics notably distinguish cavity-mediated subradiance from the exponential decay of subradiance in free space (*24*, *26*, *31*), suggesting that global metastability and abrupt reorganization are intrinsically due to the long-range, cavity-mediated forces (*32*–*34*).

We support this hypothesis by a microscopic theory that qualitatively and quantitatively aligns with the experimental results. This model allows us to show that the subradiant pattern is a fixed point of the collective dynamics. In the configuration landscape, the subradiant state is the local minimum of the selforganized potential, and the number of atoms determines the height of the barrier, as illustrated in Fig. 1D. Atom loss lowers the barrier until it reaches the stability threshold (*35*, *36*), inducing the sudden decay from the subradiant to the superradiant pattern. This transition manifests characteristics of violent relaxation, common in instabilities of astrophysical and plasma clusters (*32*, *36*–*38*), underscoring the broader relevance of strong long-range interacting systems. Our theoretical framework fully captures the nonequilibrium phase transition from the subradiant into the superradiant pattern, shedding light onto the underlying microscopic processes.

## RESULTS

### System description

The setup is composed of two high-finesse optical cavities ( ν=1,2 ) that cross under an angle of 60° [see Fig. 1 (A and B)]. A quantum gas of ^87^Rb atoms forms a Bose-Einstein condensate (BEC) of $N=2.7\left(3\right)\cdot 10^{5}$ and is placed in a dipole trap located at the crossing point of the modes. The cavities are characterized by their couplings $g_{\nu =1,2}\;/\;2\pi =\left[1.95\left(1\right),1.77\left(1\right)\right]$ MHz with the atomic dipolar transition and the field decay rates $\kappa_{\nu =1,2}\;/\;2\pi =\left[147\left(4\right),800\left(11\right)\right]$ kHz. In the plane spanned by the cavities, the atoms are illuminated by a standing wave laser at frequency $\omega_{p}$ and with Rabi frequency Ω . This pump field is far detuned by $\Delta_{a}=\omega_{p}-\omega_{a}=2\pi \times 69.8\left(1\right)$ GHz on the blue with respect to the atomic resonance frequency $\omega_{a}$ . It forms a repulsive optical lattice potential of depth $V_{p}=\Omega^{2}/\Delta_{a}$ that tends to localize the atoms at the nodes. The dispersive dynamics of the BEC-cavity system gives rise to the self-consistent formation of atomic density gratings that scatter light into the cavity and cause a shift in the cavity frequencies. The strength of these processes can be controlled by the detunings $\Delta_{\nu }=\omega_{p}-\omega_{\nu }^{c}$ between the driving laser field and the respective cavity resonance $\omega_{\nu }^{c}$ (*39*).

We do not expect cavity dissipation to play an important role in the observed dynamics. This might seem unexpected, because, in some protocols presented in this work, the bare cavity detuning $\Delta_{\nu }$ vanishes. However, the effective cavity detunings are shifted by the collective dispersive shift to ${\tilde{\Delta }}_{\nu }\approx \Delta_{\nu }-{\mathrm{NU}}_{\nu }\;/\;2$ , where $U_{\nu }=g_{\nu }^{2}\;/\;\Delta_{a}>0$ is the dispersive Stark shift per particle due to the coupling with cavity mode ν=1,2 . In the experiment, $|{\tilde{\Delta }}_{\nu }|$ is of the order of several megahertz and thus much larger than the cavity decay rates, even when the bare cavity detuning is zero, $\Delta_{\nu }=0$ . The collective dispersive shift depends on the overlap between the atomic cloud and the cavity mode, and therefore, it is a dynamical quantity. We thus present the data as a function of the bare cavity detuning $\Delta_{\nu }$.

### Stationary phase diagram

Figure 1E illustrates the stationary phase diagram as a function of the detunings $\Delta_{\nu }$ for a fixed pump strength $V_{p}$ . The white region is the normal phase where the cavity field fluctuates about the vacuum and the atoms distribute along the minima of the standing wave pump field, parallel to the gray stripes of Fig. 1C. The blue and red regions correspond to stripe patterns coherently emitting into either cavity 1 or 2, respectively. The stripe patterns are sketched in subplots A and B extending to regions where the bare pump lattice potential is maximal. These configurations are facilitated by a finite cavity field: along the stripes, cavity and laser fields destructively interfere (*40*), and the resulting potential is reduced. This mechanism is the optomechanical analog of the dynamics of localized dipoles in cavities (*15*, *41*). The emerging patterns are such that simultaneous superradiant emission into both cavities is suppressed for sufficiently low pump strengths.

**Fig. 2.**
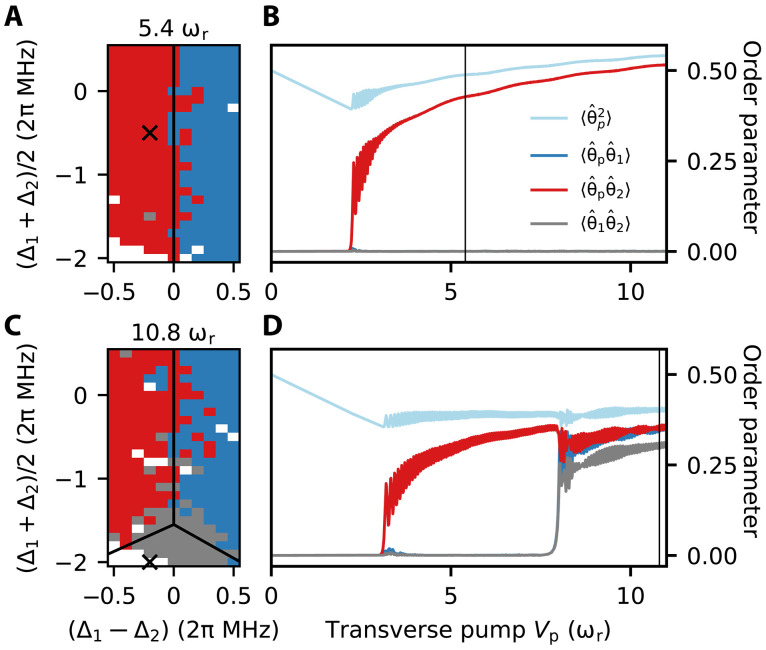
Steady-state phase diagrams and occupation of atomic modes. (**A** and **C**) Experimental steady-state phase diagrams for pump lattice depths $V_{p}=\left(5.4,10.8\right)\;\omega_{r}$ , with $\omega_{r}=2\pi \times 3.77\;\text{kHz}$ . The blue (red) color indicates light exclusively in cavity 1 (2), white indicates the absence of light, while the gray denotes coexistence, where atoms scatter in both cavities simultaneously. Black lines mark the phase boundaries calculated from the simulations of the GPE in (1). A systematic error of 2π×0.1 MHz in the cavity detunings has been taken into account (see Materials and Methods). (**B** and **D**) GPE simulations of the expectation values of the order parameters $\langle {\hat{\theta }}_{j}{\hat{\theta }}_{k}\rangle$ with j,k=p,1,2 as a function of lattice depth $V_{p}$ for the detunings indicated by crosses in (A) and (C), respectively. Vertical lines mark the lattice depths of (A) and (C), respectively. Increasing $V_{p}$ initially leads to decreasing $\langle {\hat{\theta }}_{p}^{2}\rangle$ , until self-ordering sets in and atoms are pulled toward the intensity maxima of the repulsive lattice potential. In (D), the two pump thresholds are visible: the first for self-ordering in cavity 2 and the second for self-ordering in both cavities. Note that the thresholds depend on the detunings $\Delta_{1},\Delta_{2}$.

To experimentally determine the steady-state phase diagram, we first fix the detunings $\Delta_{1}$ , $\Delta_{2}$ , and thus the expected crystalline configuration at a given lattice depth. We then linearly increase the pump lattice depth $V_{p}$ up to a target value (see Materials and Methods) and record in real time the light leaking from the cavities using two heterodyne detection setups. We report the measured phase diagrams as a function of the relative cavity detuning $\left(\Delta_{1}-\Delta_{2}\right)$ and the mean cavity detuning $\left(\Delta_{1}+\Delta_{2}\right)/2$ , which provide a convenient way to illustrate the protocols we implement in the next sections. The left panels of Fig. 2 show the measured phase diagrams for two target values of the pump amplitude. We observe three regimes: For low pump lattice depths $V_{p}$ , no self-ordering takes place, and the cavities remain empty (see Materials and Methods). For a large range of intermediate pump lattice depths, only the cavity closer to resonance with the pump is filled with light (see Fig. 2A). Last, for sufficiently deep pump lattices, a region of coexistence becomes possible, where both cavities are populated simultaneously (see the gray region in Fig. 2C).

The experimental phase diagrams are reproduced by the ground state of a modified Gross-Pitaevskii equation (GPE), for the macroscopic wave function ψ of the condensate in two dimensions

$$\begin{array}{c} i\hbar \partial_{t}\psi =\left(\;-\frac{\hbar^{2}{\vec{\nabla }}^{2}}{2m}+\sum_{\nu =x,y}\frac{1}{2}m\omega_{\nu }^{2}\nu^{2}+V_{0}N{|\;\psi \;|}^{2}+{\hat{H}}_{\text{mf}}\left[\psi \right]\;\right)\psi \end{array}$$ (1)

where ℏ is the reduced Planck constant, $V_{0}$ is the *s*-wave, contact potential, and ${\hat{H}}_{\text{mf}}$ describes the optical potential in the mean-field approximation (see Supplementary Materials for details). For blue atomic detuning, $\Delta_{a}>0$ , the optical potential can be cast into the form

$$\begin{array}{c} {\hat{H}}_{\text{mf}}\left[\psi \right]\;/\;\hbar ={\left(\;\sqrt{V_{p}}{\hat{\theta }}_{p}+\sum_{\nu =1}^{2}\sqrt{U_{\nu }}\alpha_{\nu }\left[\psi \right]{\hat{\theta }}_{\nu }\;\right)}^{2} \end{array}$$ (2)

The variables $\alpha_{\nu }\left[\psi \right]$ are the electric field amplitudes of cavity mode ν and depend on the atomic density distribution. Last, the phase-matching conditions are determined by operators ${\hat{\theta }}_{j}=\text{cos}\left(k_{j}\cdot \hat{x}\right)$ (with j=p,1,2 ) which capture the overlap between atomic density and the wave vector $k_{j}$ of the pump beam or the respective cavity. Equation 2 holds when cavity losses are negligible compared to the detuning of the dispersively shifted cavity resonance, i.e., $\kappa_{\nu }\ll \left|\Delta_{\nu }-U_{\nu }N\langle {\hat{\theta }}_{\nu }^{2}\rangle |=|{\tilde{\Delta }}_{\nu }\right|$ . Our theoretical analysis incorporates the actual cavity dissipation rates. We have also benchmarked Eq. 1 against the full master equation, finding that dissipation has negligible impact within the explored parameter regime. Therefore, we will exclusively refer to Eq. 1 in the following.

Numerical analysis and experimental data show that there are two pump threshold values for self-organization: a lower one, separating the normal phase from the phase where the gas forms stripes emitting into one cavity mode, and a second, larger threshold, at which the atoms can form checkerboard patterns that simultaneously emit into both modes. The order parameters are shown in Fig. 2 (B and D) as a function of the pump strength $V_{p}$ for two different sets of detunings. The two thresholds are visible in Fig. 2D. Here, simultaneous self-organization in both cavity modes is signaled by a finite value of the order parameter $\langle {\hat{\theta }}_{1}{\hat{\theta }}_{2}\rangle$ . Such data would be experimentally accessible via destructive time-of-flight images (*39*, *40*).

Insight into this behavior is gained from the minimization of the potential energy $E_{\text{mf}}=\langle {\hat{H}}_{\text{mf}}\rangle$ . For blue detuning, the potential energy is always positive, $E_{\text{mf}}\geq 0$ , and minimization is trivially achieved when the atoms align along the nodes of the pump lattice, illustrated by the horizontal gray stripes in Fig. 1C. This configuration becomes mechanically unstable for increasing $V_{p}$ : The new stable patterns then emit light into the cavities while minimizing the potential energy. This can occur through destructive interference between cavity fields and pump and can be summarized by the simplified condition $\sqrt{V_{p}}\geq -\left(\sqrt{U_{1}}\alpha_{1}+\sqrt{U_{2}}\alpha_{2}\right)$ for which $E_{\text{mf}}=0$ (see Supplementary Materials). This condition constrains the amplitudes of the cavity fields, setting an upper bound to the values they can take. Assume, for instance, stripes at the wave vector $k_{\text{p}}+k_{1}$ , such that $\alpha_{1}\neq 0$ and $\alpha_{2}=0$ . The constrain on the cavity field amplitude permits to estimate the maximal depth of the potential confining the stripe, ${\bar{V}}_{1}\text{cos}\left[\left(k_{\text{p}}+k_{1}\right)\cdot x\right]$ , such that it reads (see Supplementary Materials)

$${\bar{V}}_{1}=V_{p}\frac{U_{1}N}{U_{1}N\;/\;2-\Delta_{1}}$$ (3)

Mechanical stability requires that the potential height is larger than the mechanical energy transferred to an atom by photon scattering, i.e., $|\;{\bar{V}}_{\nu }\;|>C\omega_{r}$ , where C≳1 is a constant. Therefore, the stripe is (meta)stable when the pump exceeds the threshold value $V_{p}^{{\text{th}}_{\nu }}=C\omega_{r}\left(U_{\nu }N\;/\;2-\Delta_{\nu }\right)\;/\;\left(U_{\nu }N\right)$ . This argument shows that self-organization of, say, cavity 1 is favored when ${\bar{V}}_{1}>{\bar{V}}_{2}$ . In other words, the atoms self-organize in the mode with the largest dimensionless coupling strength $C_{\nu }={\mathrm{NU}}_{\nu }/{\tilde{\Delta }}_{\nu }$ . Extending these arguments, we also estimate that the threshold for simultaneous self-organization in both cavity modes (phase coexistence) is $V_{p}^{{\text{th}}_{12}}\approx 2V_{p}^{{\text{th}}_{\nu }}$ (see Supplementary Materials), which qualitatively agrees with the numerical simulation in Fig. 2D. The depth of the self-organized potential in Eq. 3 depends nonlinearly on the number of atoms.

In the rest of this work, we will focus on the parameter regime of phase exclusion, where, at equilibrium, the two types of order compete (either $\langle {\hat{\theta }}_{p}{\hat{\theta }}_{1}\rangle \neq 0$ or $\langle {\hat{\theta }}_{p}{\hat{\theta }}_{2}\rangle \neq 0$ , while $\langle {\hat{\theta }}_{1}{\hat{\theta }}_{2}\rangle =0$ ). In this regime, self-organization is expected to take place in the more strongly coupled cavity mode, and the stationary phase is determined—for constant pump intensity—by the ratio $C_{1}/C_{2}$ and thus by the experimental parameters ( $\Delta_{1},\Delta_{2}$).

### Formation of subradiant states

**Fig. 3.**
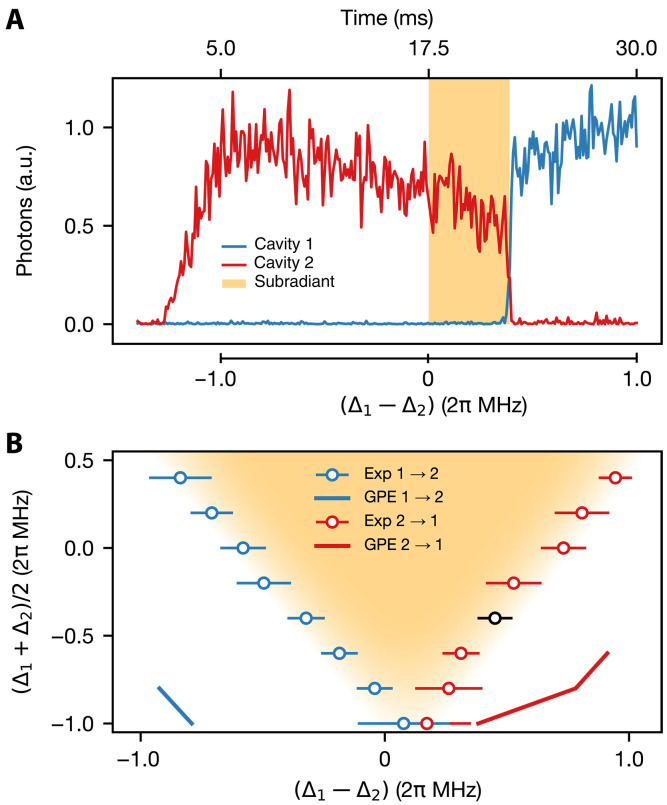
Hysteresis measurement. (**A**) Real-time evolution of the cavity occupations. In the first 5 ms, $V_{p}$ is ramped to 8.8 $\omega_{r}$ at fixed detunings. Afterward, $V_{p}$ and the mean detuning $\left(\Delta_{1}+\Delta_{2}\right)\;/\;2$ are kept constant, while the relative detuning $\left(\Delta_{1}-\Delta_{2}\right)$ is ramped in time, passing the point $\Delta_{1}=\Delta_{2}$ at 17.5 ms (see the lower axis of this plot). The cavity populations only switch past this point at a positive relative detuning, indicating hysteretic behavior (orange color), where the BEC is in the subradiant pattern. (**B**) Switching points extracted from datasets as shown in (A) as a function of the mean detuning $\left(\Delta_{1}+\Delta_{2}\right)\;/\;2$ . In the chosen parameter regime, (meta)stable coexistence does not occur. Error bars display the SD, and the orange shading indicates the metastable region of subradiance observed experimentally. The black data point indicates the detuning at which the data in (A) was taken. The solid lines show results from GPE simulations, which predict a far broader region of hysteresis than observed in the experiment. For most mean detuning values, the GPE simulation does not predict any switch out of the subradiant state within the simulated experimental runtime. a.u., arbitrary units.

Having two competing superradiant crystals allows us to explore the transition between these structures in situ. In our protocol, we keep the pump lattice depth constant and vary the cavity mode resonance frequencies, and thus the detunings $\Delta_{1}$ and $\Delta_{2}$ , as a function of time. We initialize the system in the stable superradiant state of one cavity mode, such as cavity 2, by setting $\Delta_{1}<\Delta_{2}$ (with $|\;{\tilde{\Delta }}_{1}\;|>|\;{\tilde{\Delta }}_{2}\;|$ ), by increasing the lattice depth $V_{p}$ to 8.8 $\omega_{r}$ within 5 ms, and then keeping it constant. This lattice depth is above the self-organization threshold of cavity 2 but inside the regime of phase exclusion, so that the atoms form stripes emitting superradiantly into cavity 2 while cavity 1 is empty. At t=5 ms, we linearly change the relative cavity detuning $\left(\Delta_{1}-\Delta_{2}\right)$ over 25 ms to the inverse situation, $\Delta_{1}>\Delta_{2}$ , while maintaining a constant mean detuning $\left(\Delta_{1}+\Delta_{2}\right)/2$ . A typical trace of the cavity occupations is displayed in Fig. 3A. We record the signatures of self-organization in cavity 2 during the ramp. At 5 ms, the photon number in cavity 2 starts to decay since the effective coupling $C_{2}$ decreases during the sweep of the relative detuning. From the phase diagram (Fig. 2A), one expects that the atoms should reorganize at $\Delta_{1}∼\Delta_{2}$ . Instead, we observe a pronounced hysteretic behavior, indicated by the orange shading in Fig. 3A. Only past this point of balanced coupling, the system changes emission into cavity 1 and thereby displays an increase in the photon number, following the increase of $C_{1}$ over time. In Fig. 3B, we show data for different mean detunings. The size of the hysteresis region depends monotonously on the mean cavity detuning; the stronger the atoms are effectively coupled to the cavities, the larger is the hysteresis.

This hysteretic behavior reflects the discontinuous transition between the two self-organized phases. In the bistable regime, the atoms remain stably trapped in the configuration emitting in cavity 2, despite the fact that this configuration is now excited, until the coupling to the other cavity becomes sufficiently strong. Then, at a critical value of the detuning, the cavity mode populations abruptly change, signaling a quick reorganization into the pattern that scatters superradiantly into the other cavity mode 1 and that minimizes the energy. In the metastable pattern, in particular, the atomic configuration suppresses scattering into this mode, thus inhibiting the transition into the superradiant pattern. Because of these characteristics, we identify the metastable pattern as a subradiant state.

The decay of the subradiant pattern into the superradiant phase proceeds via exponential amplification of the intracavity photon number in cavity 1. This instability reflects a nonequilibrium, driven-dissipative phase transition from subradiance to superradiance, with features reminiscent of dynamical phase transitions in systems with global-range interactions (*36*). Such subradiance-superradiance transitions have been theoretically proposed in various contexts (*42*–*44*), and our observations provide a direct manifestation of this mechanism under experimentally controlled conditions. In our case, it is implemented by dispersively coupling the cavity with the external degrees of freedom of the quantum gas by means of the pump.

The numerical simulations of this dynamics use the GPE of Eq. 1 and predict a hysteresis region that qualitatively reproduces the experimental measurement. As visible in Fig. 3B, the size of the theoretical hysteresis is however larger up to about a factor 5. We attribute this quantitative discrepancy to the model’s inability to capture several experimental features. These include noise in the detunings $\Delta_{\nu }$ , as well as a finite spatial overlap between the BEC and the two cavity mode functions, whose mode waists are located at slightly different spatial points, resulting in an inhomogeneous model and likely atomic currents during the ramp. The rates associated with contact interactions and trap oscillations, in particular, are larger than the rate of the slow ramp and are thus expected to influence the relaxation dynamics. This hypothesis is verified by comparison with numerical simulations that discard the contact interactions and the trap in the dynamics, hence setting $\omega_{\nu }=0$ and $V_{0}=0$ in Eq. 1. In this limit, the GPE is reduced to a form known as generalized GPE (GGPE) (*45*), where the sole interactions are of global range (see Supplementary Materials). Full quantitative predictions from the GGPE are limited by its high sensitivity to initial conditions, such as density fluctuations seeded during the drive ramp-up. This sensitivity, combined with the difficulty of calibrating parameters across varying mean cavity detunings, hinders direct comparison with experimental data. Nonetheless, the GGPE consistently predicts smaller hysteresis regions than observed. Since the GPE predicts substantially larger hysteresis regions than the GGPE, we identify trapping potential and interactions as stabilizing mechanisms that suppress the onset of large density fluctuations which would otherwise trigger radiative instabilities.

### Lifetime of subradiant states

**Fig. 4.**
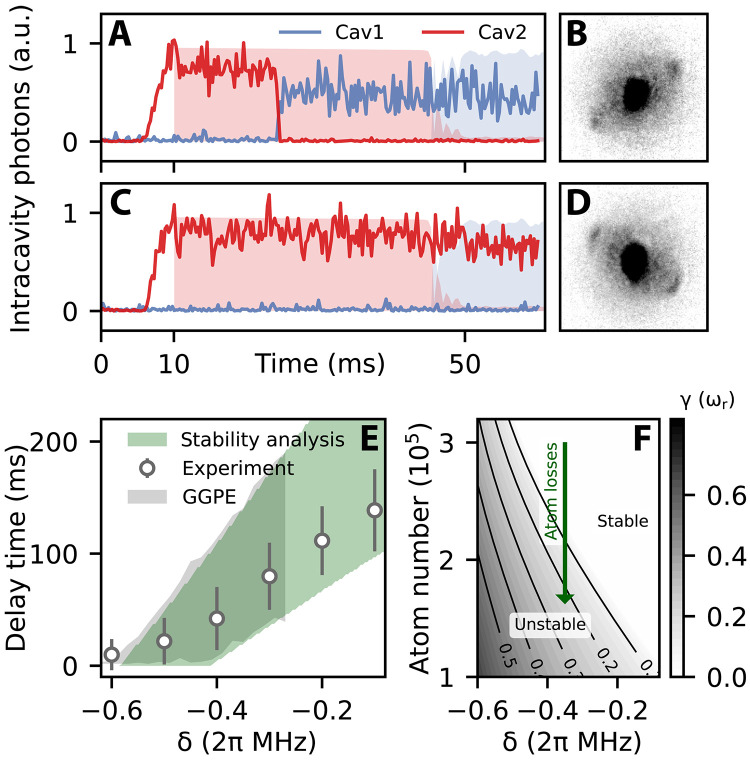
Lifetime of subradiant states. After preparing the system in a superradiant state of cavity 2 at $V_{p}=4.2\;\omega_{r}$ , the relative detuning is quenched from $\left(\Delta_{1},\Delta_{2}\right)=\left(\delta ,0\right)$ to (0,δ) . The system becomes subradiant with respect to cavity 1 until it eventually quickly reorganizes into the equilibrium, superradiant stripes with respect to cavity 1. (**A** and **C**) Two exemplary real-time traces of the cavity occupations for identical quench strength δ=2π×−0.4 MHz: For the initial 10 ms, cavity 2 is prepared in a superradiant state. After the quench, the system decays in the superradiant state of cavity 1 (A) or remains in the subradiant state for longer times (C). In the background (filled areas), we show a dynamical GGPE simulation for these parameters, initialized as superradiant state in cavity 2 at 10 ms. (**B** and **D**) Absorption images of the atomic momentum distributions at the end of the traces displayed in (A) and (C), respectively. (**E**) Delay time for which the system remains in the subradiant state, extracted from traces such as (A) and (C) as function of quench strength δ . The gray area represents the GGPE simulation results, while the green area displays the results of our stability analysis. Both theory results span from the maximum atom number (minimum atom loss) to the minimum atom number (maximum atom loss). (**F**) Escape rates out of the subradiant state derived from the stability analysis as function of relative detuning and atom number. The estimated barrier height (Eq. 3) remains constant along the solid lines. Atom loss eventually forces the system to leave the subradiant state.

Subradiant states can also be prepared by suddenly tuning the cavity detunings across the phase diagram. In this way, a stable superradiant pattern becomes an excited, subradiant state. Experimentally, we initialize the system in the superradiant state with respect to cavity 2 by ramping up the drive field within 10 ms to 4.2 $E_{r}$ at fixed detunings $\left(\Delta_{1},\Delta_{2}\right)=\left(\delta ,0\right)$ and then abruptly swap detunings between the cavities to $\left(\Delta_{1},\Delta_{2}\right)=\left(0,\delta \right)$ . Quenching the phase is analogous to extinguishing the driving field in subradiance experiments of laser-driven atoms (*24*, *25*). As shown in Fig. 4, we observe that the system keeps on emitting into cavity 2. The atoms still form the stripes that suppress emission into cavity 1, thus forming a subradiant phase. Depending on the quench parameter δ , the BEC eventually reorders after a finite time into the superradiant state, emitting light exclusively into cavity 1. We extract the lifetime of the subradiant state by fitting the time at which the cavity field occupations swap [see Fig. 4 (A and C)]. Figure 4E shows the extracted lifetimes of the subradiant states as a function of the quench strength δ.

We identify four prominent features: (i) the lifetimes exceed any dynamic timescale of the system’s constituents: The observed lifetimes are only compatible with the atom loss rate which is on the order of 3 Hz (see Supplementary Materials). (ii) The instability is characterized by a switch from the metastable subradiant to the stationary superradiant pattern, thereby preserving superfluidity, as can be seen from the absorption images in Fig. 4 (B and D). (iii) The transition itself is faster than the characteristic timescale set by trapping potential and the contact interaction. Moreover, it does not have features of nucleation. (iv) During the transition, we observe emission in both cavity modes, although the corresponding pattern of coexistence is energetically costly and thus unstable at the given pump strengths (see Fig. 2).

These features are characteristic for the out-of-equilibrium dynamics of strong long-range interacting systems. The short-time behavior of the transition exhibits the features of a violent relaxation, characteristic of Vlasov instabilities in plasmas and gravitational systems (*46*–*49*). These instabilities have a characteristic exponential amplification of fluctuations (*32*, *47*, *50*) and can be triggered by a change of the energy (*35*, *36*) or by lowering the barrier of the confining potential below the threshold (*51*, *52*). In our case, the barrier is the depth of the mechanical potential confining the stripes (see Eq. 3): The nonlinear dependence on N shows that atom losses tend to ramp it down. When the barrier is lowered to $\bar{V}∼\omega_{r}$ , the atoms flow out from the subradiant stripes at the intersection points with the superradiant stripes, filling these quickly. During this process, the atoms form a pattern that supports simultaneous emission into both cavity modes. The instability is seeded by fluctuations of the atoms’ kinetic energy in the repulsive potential. However, we are experimentally unable to identify whether the fluctuations seeding the instability are quantum or thermal. The long-lived metastable state is superfluid, and in this sense, it is an experimental example of a quantum metastable state due to long-range interactions. The reorganization process preserves superfluidity since the long-range interaction term of Eq. 1 does not affect off-diagonal long-range order.

The instability is a violent relaxation, and the process of filling the superradiant stripes via a transient excited configuration minimizes the long-range interactions. This dynamics is captured by the GGPE, which in turn describes a quantum version of the so-called generalized mean-field model (*53*). The analysis of the linear fluctuations about the equilibrium state of the GGPE shows that the subradiant phase is a fixed point and permits to describe its decay process. The linear fluctuations δψ about the subradiant state $\psi_{0}$ are governed by the equation (Supplementary Materials)

$$\hbar \omega \delta \psi =\left(\frac{\hbar^{2}{\vec{\nabla }}^{2}}{2m}-H_{\text{mf}}\left[\psi_{0}\right]\right)\delta \psi -\delta H_{\text{mf}}\left[\psi_{0}\right]\psi_{0}$$ (4)

where $H_{\text{mf}}\left[\psi_{0}\right]$ and $\delta H_{\text{mf}}\left[\psi_{0}\right]$ are, respectively, the mean-field Hamiltonian and its variation around the subradiant state, both depending on the atom number N . The instability is signaled by Im(ω)<0 , allowing us to identify a threshold value $N_{c}$ above which the subradiant state is a stable solution of the GGPE. As illustrated in Fig. 4F, it eventually becomes unstable because atom losses slowly decrease the atom number until $N<N_{c}$ . The system then quickly escapes out of the subradiant state with exponential growth at rate γ=−Im(ω) , typical of violent relaxation. The results of this analysis are reported in Fig. 4E and are in agreement with the experimental data, showing that this simplified model captures the essential features both qualitatively and quantitatively.

While Eq. 4 describes the short-time dynamics of the subradiant-superradiant transition, the full transition is captured by the GGPE. Figure 4 (A and C) shows the agreement between the photon traces of experiment and theory within the uncertainty in the atom loss rate. We have compared these predictions with the simulations using the full GPE, which again includes contact interactions and trap potentials, and found that the lifetime of the subradiant state results to be larger yet within the same order of magnitude. This confirms that the relaxation dynamics (different from the system’s response to slow ramps when measuring the hysteresis region) is primarily governed by the interplay of (quantum) fluctuations, initiating the transition, and long-range interactions, driving the system into the stable, superradiant state. In both descriptions, the final state aligns with experimental observations, demonstrating that long-range interactions dominate the overall dynamics across the subradiant-superradiant transition.

## DISCUSSION

We have investigated the relaxation dynamics of a quantum Bose gas with competing global-range interactions mediated by photons. These interactions impose two competing spatial patterns, leading to a phase diagram which we have explored by tuning the cavity frequencies. The transition between the two configurations is of first order. A slow ramp across that transition reveals a typical hysteretical behavior: Within the hysteresis region, the gas remains trapped in the pattern that suppresses emission in the other cavity, forming a subradiant grating. At the instability, it quickly reorders into the superradiant grating undergoing a nonequilibrium phase transition from a subradiant to a superradiant pattern. Energy considerations show that the reordering mechanism is dominated by the global-range interactions. Our theoretical study indicates that the breadth of the hysteresis region can be reproduced quantitatively only by taking into account fine-tuned contact interactions and spatial inhomogeneities.

The relaxation following sudden quenches across the phase diagram, instead, is solely governed by the interplay of global forces and kinetic energy. We identified parameter regimes where the subradiant patterns are excited metastable states. Their lifetime is limited only by atom losses, which slowly reduce the strength of the interactions and thus the depth of the barrier trapping the subradiant state. This dynamics is paradigmatic of collective phenomena of systems with strong long-range interactions, such as clustering, phase transitions, and fragmentation, characteristic of stars in a cluster or particles in a plasma, whose onset of stability strongly depends on the number of particles involved (*32*, *54*). Instabilities in strong long-range interacting systems due to atom losses have also been discussed in the literature of ideal models (*55*), for which our many-body cavity quantum electrodynamics (QED) setup provides a testbed.

The observed instability is seeded by density fluctuations due to the kinetic energy and exhibits characteristics of a violent relaxation, characteristic of the dynamical instability of mean-field, nonlinear equations, such as the Vlasov equation. Differing from experiments with optical systems (*56*), the instability emerges from the many-body dynamics of ultracold Bosons that interact via multiple photon scattering. Instability and reorganization in the superradiant pattern are captured by the interplay of global interactions and kinetic energy. Despite the change of energy at the transition from subradiance to superradiance, the superfluid properties of the quantum gas are preserved. The model can be mapped to a quantum realization of the paradigmatic generalized mean-field model, which predicts a plethora of nonequilibrium phase transition from the the interplay of competing long-range forces (*53*, *57*–*59*). The cavity QED dynamics thus allows to shed light on universal equilibrium and out-of-equilibrium critical phenomena due to frustration between globally interacting potentials. Our findings can also be connected to the nonequilibrium dynamics of ultracold Fermions with photon-mediated interactions in vicinity of a second-order phase transition. There, relaxation to a stationary state was observed within less than a millisecond but with typical features of a dynamical instability (*60*–*62*). Analogous dynamics have been predicted for the relaxation of thermal atoms in cavities (*63*). Such characteristics are thus distinctive of instabilities in long-range interacting systems across various length scales and in this sense universal.

Our results shed light on the individual mechanisms at work and on their interplay. By establishing a direct connection between the microscopic dynamics and the dynamical instability of a nonlinear equation for few macroscopic variables, which reproduce the experimental observations, we provide an example of universal behavior in the out-of-equilibrium dynamics. Our work demonstrates the potential of many-body cavity QED to unveil the processes leading to relaxation in long-range interacting systems. Moreover, by validating the effective nonlinear model derived for the out-of-equilibrium dynamics, this study lays the foundation for concepts and strategies to control metastable states in quantum many-body systems.

## MATERIALS AND METHODS

### Preparation of the BEC and balancing the two atom-cavity couplings

Our experimental protocol starts with the preparation of a ^87^Rb BEC of $N=2.7\left(3\right)\times 10^{5}$ and T=102(10)nK via optical evaporation. An optical dipole trap with trapping frequencies $\left[\omega_{x},\omega_{y},\omega_{z}\right]=2\pi \times \left[106\left(1\right),71.2\left(3\right),223\left(3\right)\right]\;\text{Hz}$ positions the BEC at the crossing point of the two cavity modes.

The atom-cavity coupling is proportional to the overlap of the cavity mode with the atomic cloud and can be measured via the dispersive shift of the cavity resonance. Moving the trap to different locations and measuring the dispersive shift as a function of the trap position allow us to map out the effective coupling strengths for both resonators. Since the cavity mode centers differ by 12 μm in height, where the mode waist radius is 50 μm, we can adjust the trap position such that the coupling to both resonators is balanced. Slight misalignments can however lead to a systematic shift of the phase boundary between the two ordered phases. The data in Fig. 2 has therefore been shifted by 0.1 MHz to compensate for this systematic error.

A magnetic offset field of ~25 G is applied to avoid spin-dependent effects on self-organization as, e.g., superradiance to the orthogonally polarized cavity mode that is detuned due to the birefringence of our cavity as we studied in (*64*).

### Cavities and phase diagrams

The cavity modes are tilted at an angle of 60° and have distinct decay rates $\left[\kappa_{1},\kappa_{2}\right]=2\pi \times \left[147\left(4\right),800\left(11\right)\right]\;\text{kHz}$ . The maximum single-atom vacuum Rabi frequencies of $\left[g_{1},g_{2}\right]=2\pi \times \left[1.95\left(1\right),1.77\left(1\right)\right]\text{MHz}$ are comparable for both cavities. At a 60° angle relative to each cavity, a retroreflected pump beam creates a standing wave lattice. We refer to this beam as the transverse pump. Its frequency $\omega_{p}$ is blue detuned by $\Delta_{a}=\omega_{p}-\omega_{a}=2\pi \times 69.8\left(1\right)\;\text{GHz}$ with respect to $\omega_{a}$ , the D_2_ line of ^87^Rb. We further introduce the relative detunings $\Delta_{\nu }=\omega_{p}-\omega_{\nu }^{c}$ (with ν∈{1,2} ) of the cavity resonance frequencies $\omega_{\nu }^{c}$ and the transverse pump. These detunings between the frequency of the transverse beams and the cavities are tuneable within tens of megahertz via an electro-optical modulator. The lengths of the cavities are stabilized using low-amplitude, far-detuned additional laser fields at 830 nm, allowing a constant feedback on the cavity lengths while having a negligible effect on the atomic cloud.

During each experimental sequence, we linearly increase the transverse pumping lattice depth $V_{p}$ in 10 ms from 0 to a maximum of $10.8\;\omega_{r}$ , in units of recoil frequency, $\omega_{r}=2\pi \times 3.77\;\text{kHz}$ . Meanwhile, we collect the leaking photons from each cavity and use the photon detection rates to deduce the intracavity photon numbers. Beyond a critical pump strength and for certain cavity detunings, the intracavity field is populated as the atoms self-organize into minima of the resulting lattice potential. For each cavity, we repeat the transverse pumping ramp for different cavity detunings and obtain the single cavity phase diagrams as shown in fig. S1 (A and D). The respective other cavity is not influencing this measurement since we put it to a sufficiently large detuning. The atom-cavity couplings are well balanced for both cavities, resulting in similar phase boundaries for self-organization. We further extend the individual phase diagrams to two cavities simultaneously close to resonance. By increasing the transverse pump power, we study the self-organization for different detunings of cavities 1 and 2 at a fixed transverse pump lattice strength of 5.1 $\omega_{r}$ . We plot the photon numbers in cavity 1 (2) in fig. S1 (B and E) for the given pair of cavity detunings. If the cavity field in cavity 1 (2) is larger than a defined photon number threshold, $n_{1}^{\text{th}}$ ( $n_{2}^{\text{th}}$ ), we characterize the system as self-organized in cavity 1 (2) and plot a blue (red) square in the binarized fig. S1C for the given pair of cavity detunings. A white square indicates photon levels in both cavities below the thresholds, and a gray square indicates photon levels in both cavities above the thresholds. Self-organization occurs in the cavity closer to resonance. To further highlight this dependence, we switch to a rotated coordinate system and define the relative detuning $\Delta_{1}-\Delta_{2}$ and the mean detuning ( $\Delta_{1}+\Delta_{2}$)/2 as the *x* and *y* axes (see fig. S1F).

### Heterodyne measurement

The amplitudes of the intracavity fields are recorded using balanced optical heterodyne detectors. For each cavity, a local oscillator (LO) laser field is combined at a beam splitter with the light field leaking from a cavity mirror and directed together onto the two photodiodes of a balanced photodetector. The signal is then mixed down to a moderate radio frequency of 300 kHz using a homemade IQ (in-phase and quadrature) filter device. The LO is generated by shifting the frequency of the same laser source as the transverse pump by 50 MHz with an acousto-optic modulator. To compensate for phase drifts introduced by sending the light fields through separate optical fibers, we lock their relative phase after passing through the fibers. For each cavity signal, the 300-kHz data are recorded using a computer connected oscilloscope (PicoScope 5444b). The signal is then processed by software which extracts the photon level and applies a low-pass filter using a binning window of 1 × 10^−4^ s. All associated radio frequency signal sources are phase-locked to a 10-MHz GPS frequency standard which has a fractional stability better than 10^−12^ to minimize the technical phase noise in the heterodyne detection system. Further technical details and design considerations are described in detail in (*65*). To calibrate the amplitude of the intracavity light field, we perform Raman-Nath diffraction analogous to the transverse pump calibration by applying a short coherent on-axis probe to the cavity.

### Imaging of the atomic cloud

We use resonance absorption imaging of the atomic cloud in addition to the detection of the light field leaking from the cavities. From absorption images taken after 20 ms of ballistic expansion of the atomic cloud, the temperature, number of atoms, and momentum state populations are extracted.

## Supplementary Materials

### This PDF file includes:

Supplementary Text Figs. S1 and S2 References
