# Supplementary material for: Stability and decay of subradiant patterns in a quantum gas with photon-mediated interactions
Source: Sci Adv. 2025 Jul 16;11(29):eadw0299. doi: 10.1126/sciadv.adw0299 (PMC13155537; doi:10.1126/sciadv.adw0299)
Supplement: Supplementary file 1 — Supplementary Text Figs. S1 and S2 References [file sciadv.adw0299_sm.pdf]

Science Advances
AAAS

Supplementary Materials for

Stability and decay of subradiant patterns in a quantum gas with photon-mediated interactions

Alexander Baumgärtner *et al.*

Corresponding author: Tobias Donner, donner@phys.ethz.ch

Sci. Adv. **11**, eadw0299 (2025)
DOI: 10.1126/sciadv.adw0299

This PDF file includes:

Supplementary Text
Figs. S1 and S2
References

Theoretical Model: Gross-Pitaevskii equation with global-range interactions

In this Appendix, we provide details about the theoretical model of the system dynamics and the numerical simulations we performed, leading to the theoretical data presented in the main text. We first introduce the full quantum mechanical model of atoms and cavities. By adiabatically eliminating the cavities' degrees of freedom and employing a mean-field ansatz for the state of the atomic sector, we derive in the subsequent sections the mean-field Hamiltonian of Eq. (2) in the main text. At the end, we give additional information about the numerical simulation of the GPE, Eq. (1) of the main text, for the various protocols of the experiment.

Quantum master equation for the composite system

In what follows, we denote by $\hat{a}_\nu$ and $\hat{a}_\nu^\dagger$ the annihilation and creation operators of cavity mode ν at frequency ω_ν^c and wave vector $\boldsymbol{k}_\nu$, obeying the bosonic commutation relations $[\hat{a}_\nu, \hat{a}_\mu^\dagger] = \delta_{\nu,\mu}$ and $[\hat{a}_\nu, \hat{a}_\mu] = 0$. For convenience, we treat the atoms in first quantization and denote by $\hat{\boldsymbol{x}}_j$ and $\hat{\boldsymbol{p}}_j$ the canonically-conjugate positions and momenta in the $x-y$ plane. A given atoms' dipolar transition at frequency ω_a couples with both cavity modes and with a transverse classical field at frequency ω_p and wave vector $\boldsymbol{k}_p$. Below we assume that the detuning $\Delta_a = \omega_p - \omega_a$ of the laser from the atomic transition is the largest frequency, allowing us to eliminate the atoms' internal degrees of freedom from the equation of cavity and atomic external degrees of freedom and to discard spontaneous emission *(66)* . This requires that the spontaneous decay rate $\gamma \ll |\Delta_a|$. It further requires that the laser Rabi frequency $\Omega \ll |\Delta_a|$ and that the vacuum Rabi frequencies satisfy $g_\nu\sqrt{N} \ll |\Delta_a|$. As in the experiment, we will take $\Delta_a > 0$. After eliminating the electronic degrees of freedom, we obtain the quantum master equation governing the dynamics of the density operator $\hat{\rho}$, which describes the state of the two cavity modes ($\nu = 1, 2$) and of the motional degrees of freedom of the particles in the reference frame rotating at the laser

frequency ω_p *(40, 66)* :

$$\frac{\partial}{\partial t}\hat{\rho} = -\frac{i}{\hbar}\left[\hat{H},\hat{\rho}\right] + \sum_{\nu=1}^{2}\kappa_\nu \mathcal{L}[\hat{a}_\nu]\hat{\rho}\,. \tag{S1}$$

The cavity modes' losses at rate κ_ν are accounted for by the last term, where $\mathcal{L}[\hat{a}_\nu]\hat{\rho} = 2\hat{a}_\nu\hat{\rho}\hat{a}_\nu^\dagger - \hat{a}_\nu^\dagger\hat{a}_\nu\hat{\rho} - \hat{\rho}\hat{a}_\nu^\dagger\hat{a}_\nu$. The (effective) Hamiltonian $\hat{H} = \hat{H}_\mathrm{a} + \hat{H}_\mathrm{f} + \hat{V}_\mathrm{int}$ is the sum of the coherent free dynamics of the N particles ($\hat{H}_\mathrm{a}$), of the cavity modes ($\hat{H}_\mathrm{f}$), and of their optomechanical interactions $\hat{V}_\mathrm{int}$:

$$\hat{H}_\mathrm{a} = \sum_{j=1}^{N}\left(\frac{\hat{\boldsymbol{p}}_j^2}{2m} + \hbar V_p\cos^2(\boldsymbol{k}_p\cdot\hat{\boldsymbol{x}}_j)\right)\,, \tag{S2}$$

$$\hat{H}_\mathrm{f} = -\sum_{\nu=1}^{2}\hbar\Delta_\nu\hat{a}_\nu^\dagger\hat{a}_\nu\,, \tag{S3}$$

$$\hat{V}_\mathrm{int} = \sum_{\nu=1}^{2}\hbar U_\nu\hat{\Theta}_{\nu\nu}\hat{a}_\nu^\dagger\hat{a}_\nu + \sum_{\nu=1}^{2}\hbar\sqrt{V_pU_\nu}\hat{\Theta}_{p\nu}(\hat{a}_\nu + \hat{a}_\nu^\dagger) + \hbar\sqrt{U_1U_2}\hat{\Theta}_{12}(\hat{a}_1^\dagger\hat{a}_2 + \hat{a}_2^\dagger\hat{a}_1)\,, \tag{S4}$$

where $V_p = \Omega^2/\Delta_a$ is the pump lattice depth and $U_\nu = g_\nu^2/\Delta_a$ is the single-particle dispersive shift due to the coupling with the cavity mode *(18)* . We used the atomic operators

$$\hat{\Theta}_{\mu\nu} = \sum_{j=1}^{N}\cos(\boldsymbol{k}_\mu\cdot\hat{\boldsymbol{x}}_j)\cos(\boldsymbol{k}_\nu\cdot\hat{\boldsymbol{x}}_j)\,, \quad \mu,\nu = p,1,2\,, \tag{S5}$$

where $\boldsymbol{k}_1$ and $\boldsymbol{k}_2$ are the wavevectors of the cavities. The slow experimental ramps of laser intensity and the cavity frequencies are described by treating V_p and Δ_ν as time-dependent coefficients in (S1).

Adiabatic elimination of the cavities degrees of freedom

In order to simplify the theoretical description of the system, we employ the method developed in Refs. *(67)* to adiabatically eliminate the cavities' degrees of freedom from the master equation (S1). This leads to an effective master equation, which we here report for the specific choice $\Delta_a > 0$:

$$\frac{\partial}{\partial t}\hat{\rho}_\mathrm{sys} = -\frac{i}{\hbar}\left[\hat{H}_\mathrm{a} + \frac{\hbar}{2}\sum_{\nu=1}^{2}\sqrt{V_pU_\nu}\left(\hat{\alpha}_\nu^\dagger\hat{\Theta}_{p\nu} + \hat{\Theta}_{p\nu}^\dagger\hat{\alpha}_\nu\right), \hat{\rho}_\mathrm{sys}\right] + \sum_{\nu=1}^{2}\kappa_\nu\mathcal{L}[\hat{\alpha}_\nu]\hat{\rho}_\mathrm{sys}\,, \tag{S6}$$

describing the dynamics of the particles' external degrees of freedom, with $\hat{\rho}_{\rm sys}$ the reduced atomic density operator. The atomic operators $\hat{\alpha}_{1,2}$ represent effective quantum fields *(68)* and they obey the coupled equations of motion

$$\frac{\partial}{\partial t}\hat{\alpha}_1 = -\frac{i}{\hbar}[\hat{H}_{\rm a}, \hat{\alpha}_1] - i\hat{Z}_1\hat{\alpha}_1 - i\sqrt{V_pU_1}\hat{\Theta}_{p1} - i\sqrt{U_1U_2}\hat{\Theta}_{12}\hat{\alpha}_2\,, \tag{S7}$$

$$\frac{\partial}{\partial t}\hat{\alpha}_2 = -\frac{i}{\hbar}[\hat{H}_{\rm a}, \hat{\alpha}_2] - i\hat{Z}_2\hat{\alpha}_2 - i\sqrt{V_pU_2}\hat{\Theta}_{p2} - i\sqrt{U_1U_2}\hat{\Theta}_{12}\hat{\alpha}_1\,. \tag{S8}$$

When the time scales of the cavity mode dynamics are much shorter than the ones characterizing the motion, we can replace the operators $\hat{\alpha}_{1,2}$ in (S6) by their adiabatic solutions, see Refs *(66, 68)*. The latter are found from the previous equations by setting $\partial_t\hat{\alpha}_{1,2} = 0$ and neglecting the kinetic energy during the cavity relaxation to the local steady state, and take the form:

$$\hat{\alpha}_1 = \sqrt{V_pU_1}\frac{\hat{\Theta}_{p1}\hat{Z}_2 - U_2\hat{\Theta}_{12}\hat{\Theta}_{p2}}{U_1U_2\hat{\Theta}_{12}^2 - \hat{Z}_1\hat{Z}_2} \quad \text{and} \quad \hat{\alpha}_2 = \sqrt{V_pU_2}\frac{\hat{\Theta}_{p2}\hat{Z}_1 - U_1\hat{\Theta}_{12}\hat{\Theta}_{p1}}{U_1U_2\hat{\Theta}_{12}^2 - \hat{Z}_1\hat{Z}_2}\,, \tag{S9}$$

where $\hat{Z}_\nu = -\Delta_\nu + U_\nu\hat{\Theta}_{\nu\nu} - i\kappa_\nu$, with $\nu = 1, 2$.

Mean-field Hamiltonian

The mean-field master equation is derived using the mean-field ansatz

$$\hat{\rho}_{\rm sys} = \otimes_{j=1}^N \hat{\rho}_a\,. \tag{S10}$$

Integrating out the other $N-1$ variables we obtain the (non-linear) mean-field master equation

$$\frac{\partial}{\partial t}\hat{\rho}_a = -\frac{i}{\hbar}\left[\frac{\hat{\boldsymbol{p}}^2}{2m} + \hat{H}_{\rm mf}[\hat{\rho}_a], \hat{\rho}_a\right]\,, \tag{S11}$$

describing the dynamics of the single-particle density operator $\hat{\rho}_a$. The mean-field Hamiltonian takes the form

$$\hat{H}_{\rm mf}[\hat{\rho}_a] = \hbar V_p\hat{\theta}_p^2 + \sum_{\nu=1}^{2}\hbar U_\nu|\alpha_\nu[\hat{\rho}_a]|^2\hat{\theta}_\nu^2 + \sum_{\nu=1}^{2}2\hbar\sqrt{V_pU_\nu}\mathrm{Re}(\alpha_\nu[\hat{\rho}_a])\hat{\theta}_p\hat{\theta}_\nu + 2\hbar\sqrt{U_1U_2}\mathrm{Re}(\alpha_1^*[\hat{\rho}_a]\alpha_2[\hat{\rho}_a])\hat{\theta}_1\hat{\theta}_2\,. \tag{S12}$$

Here, $\hat{\theta}_\mu = \cos(\boldsymbol{k}_\mu \cdot \hat{\boldsymbol{x}})$, with $\mu = p, 1, 2$, and the amplitudes of the cavity fields $\alpha_{1,2} = \langle\hat{\alpha}_{1,2}\rangle \equiv \mathrm{Tr}\{\hat{\alpha}_{1,2}\hat{\rho}_{\mathrm{sys}}\}$, with the operators $\hat{\Theta}_{\mu\nu}$ essentially replaced by the expectation values $N\langle\hat{\theta}_\mu\hat{\theta}_\nu\rangle$. Their expressions are found by using the steady-state equations (S9) and using the mean-field approximation $\langle\hat{\Theta}_{\mu\nu}\hat{\alpha}_{1,2}\rangle \approx N\langle\hat{\theta}_\mu\hat{\theta}_\nu\rangle\langle\hat{\alpha}_{1,2}\rangle$:

$$\begin{aligned}\alpha_1[\hat{\rho}_a] &= \sqrt{V_p U_1} N \frac{\langle\hat{\theta}_p\hat{\theta}_1\rangle Z_2 - U_2 N \langle\hat{\theta}_p\hat{\theta}_2\rangle\langle\hat{\theta}_1\hat{\theta}_2\rangle}{U_1 U_2 N^2 \langle\hat{\theta}_1\hat{\theta}_2\rangle^2 - Z_1 Z_2} \quad \text{and} \\ \alpha_2[\hat{\rho}_a] &= \sqrt{V_p U_2} N \frac{\langle\hat{\theta}_p\hat{\theta}_2\rangle Z_1 - U_1 N \langle\hat{\theta}_p\hat{\theta}_1\rangle\langle\hat{\theta}_1\hat{\theta}_2\rangle}{U_1 U_2 N^2 \langle\hat{\theta}_1\hat{\theta}_2\rangle^2 - Z_1 Z_2}\,,\end{aligned} \tag{S13}$$

where $Z_{\nu=1,2} = U_\nu N\langle\hat{\theta}_\nu^2\rangle - \Delta_\nu - i\kappa_\nu$. Hamiltonian $\hat{H}_{\mathrm{mf}}$ depends nonlinearly on the atomic state through the functionals $\alpha_{1,2}$. The latter can be rewritten as

$$\alpha_1[\hat{\rho}_a] \;=\; -\frac{N\sqrt{V_p U_1}}{Z_1} \frac{\langle\hat{\theta}_p\hat{\theta}_1\rangle + C_2'\langle\hat{\theta}_p\hat{\theta}_2\rangle\langle\hat{\theta}_1\hat{\theta}_2\rangle}{1 - C_1' C_2' \langle\hat{\theta}_1\hat{\theta}_2\rangle^2}\,, \tag{S14}$$

where $C_\nu' = -NU_\nu/Z_\nu$. Note that $C_\nu' \to C_\nu$ for negligible cavity decay rate.

For vanishing cavity loss rates (namely, when α_ν is real), the mean-field Hamiltonian (S12) can be cast in the form

$$\hat{H}_{\mathrm{mf}}^{(\mathrm{blue})} \;=\; \hbar\left(\sqrt{V_p}\hat{\theta}_p + \sum_{\nu=1}^{2}\sqrt{U_\nu}\alpha_\nu\hat{\theta}_\nu\right)^2\,, \tag{S15}$$

thus showing that the mean-field energy is bounded from below, $\langle\hat{H}_{\mathrm{mf}}^{(\mathrm{blue})}\rangle \geq 0$. This expression is strictly valid provided that V_p, $U_{\nu=1,2}$ are positive, corresponding to an atom-pump detuning on the blue side, $\Delta_{\mathrm{a}} > 0$. For red detunings, instead, these quantities are negative and the mean-field Hamiltonian can be rewritten as

$$\hat{H}_{\mathrm{mf}}^{(\mathrm{red})} \;=\; -\hbar\left(\sqrt{|V_p|}\hat{\theta}_p + \sum_{\nu=1}^{2}\sqrt{|U_\nu|}\alpha_\nu\hat{\theta}_\nu\right)^2. \tag{S16}$$

Blue self-organization, metastability, and threshold for phase coexistence

Opposite to the red-detuned case, where the mean-field energy is always negative, for blue detuning the mean-field energy is positive. This is an essential feature, that leads to strikingly

different equilibrium properties. Self-organization in the blue takes place within the detuning range $\Delta_\nu \in [-NU_\nu/2, NU_\nu/2]$, where the specific size of the interval varies as a function of V_p *(40)*. In addition, when the two cavity mode wave vectors are neither parallel nor orthogonal, we observe that simultaneous self-organization in both modes (phase coexistence) occurs at a larger pump threshold than for self-organization in one of the modes, even for $\Delta_1 \approx \Delta_2$. This is also in contrast with self-organization for red detunings, where the different orders typically coexist and can even mutually enhance each other *(39)*. Below we provide a simple argument for the existence of two thresholds for the pump, a lower one, $V_p^{\mathrm{th}_\nu}$, separating the disordered phase from self-organization in one mode and a higher one,$V_p^{\mathrm{th}_{12}}$, separating single-mode ordering from phase coexistence.

Minimization of the mean-field energy, $\langle \hat{H}_{\mathrm{mf}}^{(\mathrm{blue})} \rangle = 0$, is trivially achieved when the atoms are at the nodes of the pump lattice and the cavity modes are empty. This becomes mechanically unstable for increasing V_p, and the new stationary state is characterized by patterns such that one or both cavity fields do not vanish but destructively interfere with the pump field at the atomic positions. For blue-detuned self-organization, we can assume a maximal overlap of $1/2$ between the atomic density and any of the potentials, $\langle \hat{\theta}^2_{\nu=1,2} \rangle = \langle \hat{\theta}^2_p \rangle = \langle \hat{\theta}_\nu \hat{\theta}_p \rangle = 1/2$, since density stripes can form in the direction determined by either $\boldsymbol{k}_p + \boldsymbol{k}_\nu$ or $\boldsymbol{k}_p - \boldsymbol{k}_\nu$. Specifically, also phase coexistence requires $\langle \hat{\theta}_1 \hat{\theta}_2 \rangle = 1/2$. Using these relations, the mean-field energy vanishes when the amplitudes of the cavity field modes satisfy the relation:

$$\sqrt{V_p} = -\left(\sqrt{U_1}\alpha_1 + \sqrt{U_2}\alpha_2\right) , \tag{S17}$$

which can be exactly fulfilled for $NU_\nu \gg |\Delta_\nu|$ *(41)*.

We first determine the threshold $V_p^{\mathrm{th}_\nu}$ on the pump for self-organization in one cavity mode and assume that the atoms form stripes at the wave vector $\boldsymbol{k}_p + \boldsymbol{k}_1$, such that $\alpha_1 = -\sqrt{V_p/U_1}$ and $\alpha_2 = 0$. The stripes are confined by the potential $\bar{V}\cos((\boldsymbol{k}_p + \boldsymbol{k}_1)\cdot \boldsymbol{x})$, with depth $\bar{V} =$

$\sqrt{V_p U_1}\alpha_1$, and are mechanically stable when the potential height is larger than the minimal amount of mechanical energy transferred to an atom by photon scattering, i.e. when $|\bar{V}| > \mathcal{C}\omega_r$, where $\mathcal{C} \gtrsim 1$ is a constant. Therefore, the stripes' mechanical stability requires $|\alpha_1| \geq \mathcal{C}\omega_r/\sqrt{V_p U_1}$. The explicit dependence of the amplitude α_1 on V_p is given in (S14). For $\alpha_2 = 0$ then

$$\bar{V} = V_p \frac{U_1 N}{U_1 N/2 - \Delta_1} \,. \tag{S18}$$

and the stripe is (meta)stable when the pump exceeds the threshold value $V_p^{\mathrm{th}_\nu} = V_p^{\mathrm{th}_1}$, with $V_p^{\mathrm{th}_1} \equiv \mathcal{C}\omega_r(U_1 N/2 - \Delta_1)/(U_1 N)$ a function of the detuning Δ_1. Note that $V_p^{\mathrm{th}_1} \leq \mathcal{C}\omega_r$. The depth of the potential confining the stripes, $\bar{V}$, is also the barrier that separates the subradiant from the superradiant pattern. The subradiant pattern, in particular, becomes unstable when the number of atoms reaches the value such that $\bar{V} \leq \mathcal{C}\omega_r$.

Relation (S17) leads to a constraint for the maximum value that each field amplitude can reach, $|\alpha_\nu| \leq \sqrt{V_p/U_\nu}$, see Ref *(41)*, and constrains at the same time the maximum value of the second cavity field mode, given a certain field in the first cavity. This allows us to estimate a lower bound $V_p^{\mathrm{th}_{12}}$ for the pump threshold for phase coexistence. Starting from (S17), self-organization in both cavity modes requires that both (negative) cavity field amplitudes satisfy $|\alpha_\nu| > \mathcal{C}\omega_r/\sqrt{U_\nu V_p}$, leading to the inequality $\sqrt{V_p} \geq 2\mathcal{C}\omega_r/\sqrt{V_p}$. The value of $V_p = V_p^{\mathrm{th}_{12}}$ at which the equality holds is the threshold for phase coexistence, and reads:

$$V_p^{\mathrm{th}_{12}} = 2\mathcal{C}\omega_r \,. \tag{S19}$$

This is at least twice as large as the threshold for stripe formation $V_p^{\mathrm{th}_\nu}$ and qualitatively agrees with the numerical simulation in Fig. 2**D**.

Gross-Pitaevskii and Generalized Gross-Pitaevskii equation

The Gross-Pitaevskii equation (GPE), Eq. (1) in the main text, is derived from (S12) after writing the mean-field Hamiltonian in second quantization, adding the trapping potential and the

van-der-Waals scattering term, and making the ansatz of symmetry breaking. The generalized Gross-Pitaevskii equation (GGPE) follows directly from the GPE by neglecting the trapping potential, $\omega_{\nu=x,y} = 0$, and the short-range interaction, $V_0 = 0$:

$$i\hbar\frac{\partial}{\partial t}\psi(\boldsymbol{x},t) = \left(-\frac{\hbar^2\nabla^2}{2m} + H_{\mathrm{mf}}[\psi]\right)\psi(\boldsymbol{x},t)\,. \tag{S20}$$

The mean-field Hamiltonian H_{mf} is identical to (S12), where the expectation values in the cavity amplitudes, (S13), are now taken over the wavefunction ψ, $\langle\hat{\theta}_\mu\hat{\theta}_\nu\rangle = \int \mathrm{d}\boldsymbol{x}\ \psi^*(\boldsymbol{x},t)\cos(\boldsymbol{k}_\mu\cdot\boldsymbol{x})\cos(\boldsymbol{k}_\nu\cdot\boldsymbol{x})\psi(\boldsymbol{x},t)$.

Numerical Simulations

In this section, we give further details on the numerical simulations we performed based on the GPE and the GGPE.

Implementation

The GPE has been implemented numerically using the Julia package QuantumOptic.jl *(69)*. The wavefunction $\psi(\boldsymbol{x},t)$ is here discretized in space over a two-dimensional grid $[-L_x/2, L_x/2]\times[-L_y/2, L_y/2]$ with $n_x \times n_y$ points. The lengths $L_{x,y}$ are fixed by the extension of the atomic cloud, which can be estimated by the spatial extension of a trapped two-dimensional BEC in its ground state. In the Thomas-Fermi limit, the ground-state density is close to the Thomas-Fermi solution $|\psi_{\mathrm{TF}}|^2$, found by solving the GPE (see Eq. (1) of the main text) for its steady state when neglecting the mean-field potential H_{mf} and the kinetic energy term:

$$|\psi_{\mathrm{TF}}(x,y)|^2 = \frac{2}{\pi R_x R_y}\begin{cases}1-\left(\frac{x}{R_x}\right)^2-\left(\frac{y}{R_y}\right)^2, & (x/R_x)^2+(y/R_y)^2\leq 1\\ 0, & \text{otherwise}\,.\end{cases} \tag{S21}$$

The size of the cloud is characterized by the Thomas-Fermi radii

$$R_\nu = \frac{1}{\omega_\nu}\left(\frac{4}{m\pi}V_0 N\omega_x\omega_y\right)^{\frac{1}{4}}\,,\quad \nu = x,y\,. \tag{S22}$$

Typical grid sizes $L_{x,y}$ are chosen between $1.4 - 1.6$ times the Thomas-Fermi radii. While the extension of the cloud is mainly dictated by the short-range interaction and the harmonic trapping potential, the step sizes L_x/n_x and L_y/n_y are controlled by the light fields of the pump and the cavities, in particular, the periodic potentials they create. For the considered values of the cavity detunings $\Delta_{1,2}$, we can assume that all potentials are chracterized by the same wavenumber $k = \sqrt{2m\omega_r/\hbar} \approx 8\ \mu\mathrm{m}^{-1}$, with ω_r the recoil frequency. In the simulations, we choose the stepsizes to fit $8 - 12$ grid points within one wavelength $\lambda = 2\pi/k$ to resolve these structures. For the experimental parameters, this requires typically a grid of $n_{x,y} = 200$ - 400 points per spatial direction.

It is worth noting that for solving the GGPE of (S20), we can exploit the periodicity of the mean-field Hamiltonian (S12) and reduce the spatial grid to a single unit cell, as confirmed by the numerics. The unit cell is determined by the wavevectors of the pump and cavity modes, given for the specific geometry of our experimental setup by $\boldsymbol{k}_p = k\boldsymbol{e}_y$, $\boldsymbol{k}_1 = k(\sqrt{3}/2\boldsymbol{e}_x - 1/2\boldsymbol{e}_y)$, and $\boldsymbol{k}_2 = k(\sqrt{3}/2\boldsymbol{e}_x + 1/2\boldsymbol{e}_y)$. Here, $\boldsymbol{e}_x$ and $\boldsymbol{e}_y$ are the unit vectors of the two spatial directions and, without loss of generality, we align the y-axis with the pump axis. The dimensions of the unit cell are here $L_x = (4\pi/\sqrt{3})k^{-1}$ and $L_y = 4\pi k^{-1}$.

Contact-interaction potential

In three dimensions, the contact interaction is given by $V_0^{(3\mathrm{D})} = 4\pi\hbar^2 a_\mathrm{s}/m$ *(73)*. For Rubidium 87, the mass is $m = 1.44316 \times 10^{-25}$ kg and the scattering length is $a_\mathrm{s} = 98a_0$, with a_0 the Bohr radius. In three dimensions, the Thomas-Fermi radii can be derived similarly to the preceding paragraph, leading to

$$R_\nu^{(3\mathrm{D})} = \frac{1}{\omega_\nu}\left(\frac{15}{4\pi}\frac{V_0^{(3\mathrm{D})}N\omega_x\omega_y\omega_z}{m}\right)^{\frac{1}{5}} . \tag{S23}$$

The two-dimensional contact interaction strength V_0 in our GPE simulations is chosen so that the extension of the cloud in the x-y-plane matches the one of a three-dimensional cloud. Thus,

by requiring that $R_{x,y}^{(3D)} = R_{x,y}$, we find the following expression for the two-dimensional contact interaction strength

$$V_0 = \left(\frac{15^4 \pi}{2^{18}} \frac{m\omega_z^4}{\omega_x \omega_y} \frac{\left(V_0^{(3D)}\right)^4}{N} \right)^{\frac{1}{5}} . \tag{S24}$$

Normalization and atom losses

The condensate wavefunction ψ is here defined such that the normalization $\mathcal{P} = \int \mathrm{d}\boldsymbol{x} \mid \psi(\boldsymbol{x})|^2 = 1$ is independent of the number of particles N, as such, N appears explicitly in the GPE via the short- and long-range interactions (see, for instance, (S13)). When solving the GPE for the dynamics of the system, we account for the atom losses of the experiment. This is done by adding to the GPE a phenomenological loss term $-i\Gamma/2\psi$, leading to an exponential decay of the initially normalized wavefunction over time with rate Γ, such that $\mathcal{P}(t) = e^{-\Gamma t}$. As extracted from the characterization of the experiment (see Sec. "Atomic loss rate"), we estimate the loss rate Γ to range between 2 and 4 Hz. In the presence of atom losses, N represents in the dynamical equation the initial number of atoms in the BEC, while the number of atoms at time t is given by $N\mathcal{P}(t)$. It is important to note that the contact interaction strength (S24) has to be replaced by $V_0 e^{\Gamma t/5}$ in the GPE to ensure the correct size of the cloud in the x-y-plane (see Sec. "Contact-interaction potential") at any time t.

Linear Stability Analysis

In this Section, we present the details of the linear stability analysis used in Fig. (4) of the main text to determine the lifetime of the subradiant states.

Determining the stability of the fixed points

The fixed points ψ_0 ($\partial_t\psi_0 = 0$) of the GGPE (S20), including the subradiant states, obey the equation

$$\left(-\frac{\hbar^2\nabla^2}{2m} + H_{\mathrm{mf}}[\psi_0]\right)\psi_0 = 0\,. \tag{S25}$$

In order to determine whether a fixed point is stable, one needs to perform a linear stability analysis. For a detailed discussion of this method, we refer the reader to textbooks about non-linear dynamics, such as Ref *(71)* and references therein.

The stability of ψ_0 can be determined by analyzing the dynamics of small fluctuations $\delta\psi(\boldsymbol{x},t) = \psi(\boldsymbol{x},t) - \psi_0(\boldsymbol{x})$ about the fixed point. As follows from the GGPE, the fluctuation obeys the exact equation of motion

$$i\hbar\frac{\partial}{\partial t}\delta\psi(\boldsymbol{x},t) = \left(-\frac{\hbar^2\nabla^2}{2m} + H_{\mathrm{mf}}[\psi_0+\delta\psi]\right)\left(\psi_0(\boldsymbol{x}) + \delta\psi(\boldsymbol{x},t)\right)\,. \tag{S26}$$

The fluctuations give rise to small variations of the observables' expectation values about their steady value $\langle\theta_\mu\theta_\nu\rangle_0 = \int \mathrm{d}\boldsymbol{x}\ \psi_0^*(\boldsymbol{x})\theta_\mu(\boldsymbol{x})\theta_\nu(\boldsymbol{x})\psi_0(\boldsymbol{x})$ *(75)*, where $\theta_\mu(\boldsymbol{x}) = \cos(\boldsymbol{k}_\mu\cdot\boldsymbol{x})$. We now use perturbation theory in first order in those variations, hereafter denoted by

$$\langle\theta_\mu\theta_\nu\rangle_{\delta\psi} = \int \mathrm{d}\boldsymbol{x}\ \theta_\mu(\boldsymbol{x})\theta_\nu(\boldsymbol{x})\left(\delta\psi^*(\boldsymbol{x},t)\psi_0(\boldsymbol{x}) + \psi_0^*(\boldsymbol{x})\delta\psi(\boldsymbol{x},t)\right)\,. \tag{S27}$$

The cavity amplitude $\alpha_1[\psi_0+\delta\psi]$ to first order in the variations reads

$$\alpha_1[\psi_0+\delta\psi] = \alpha_1[\psi_0] + \delta\alpha_1\,, \tag{S28}$$

with

$$\begin{aligned}\delta\alpha_1 =&a_{1,11}[\psi_0]\langle\theta_1^2\rangle_{\delta\psi} + a_{1,22}[\psi_0]\langle\theta_2^2\rangle_{\delta\psi} + a_{1,p1}[\psi_0]\langle\theta_p\theta_1\rangle_{\delta\psi} +\\ &+ a_{1,p2}[\psi_0]\langle\theta_p\theta_2\rangle_{\delta\psi} + a_{1,12}[\psi_0]\langle\theta_1\theta_2\rangle_{\delta\psi}\,.\end{aligned} \tag{S29}$$

The expansion coefficients are given by

$$
\begin{aligned}
a_{1,11}[\psi_0] &= \frac{U_1 N Z_{2,\mathrm{ss}}}{U_1 U_2 N^2 \langle\theta_1\theta_2\rangle_0^2 - Z_{1,\mathrm{ss}} Z_{2,\mathrm{ss}}} \alpha_1[\psi_0]\,, \\
a_{1,22}[\psi_0] &= \frac{U_2 N \left(\sqrt{V_p U_1} N \langle\theta_p\theta_1\rangle_0 + Z_{1,\mathrm{ss}}\alpha_1[\psi_0]\right)}{U_1 U_2 N^2 \langle\theta_1\theta_2\rangle_0^2 - Z_{1,\mathrm{ss}} Z_{2,\mathrm{ss}}}\,, \\
a_{1,p1}[\psi_0] &= \frac{\sqrt{V_p U_1} N Z_{2,\mathrm{ss}}}{U_1 U_2 N^2 \langle\theta_1\theta_2\rangle_0^2 - Z_{1,\mathrm{ss}} Z_{2,\mathrm{ss}}}\,, \qquad \text{(S30)} \\
a_{1,p2}[\psi_0] &= -\frac{U_2 N \sqrt{V_p U_1} N \langle\theta_1\theta_2\rangle_0}{U_1 U_2 N^2 \langle\theta_1\theta_2\rangle_0^2 - Z_{1,\mathrm{ss}} Z_{2,\mathrm{ss}}}\,, \\
a_{1,12}[\psi_0] &= -\frac{U_2 N \left(\sqrt{V_p U_1} N \langle\theta_p\theta_2\rangle_0 + 2U_1 N \langle\theta_1\theta_2\rangle_0 \alpha_1[\psi_0]\right)}{U_1 U_2 N^2 \langle\theta_1\theta_2\rangle_0^2 - Z_{1,\mathrm{ss}} Z_{2,\mathrm{ss}}}\,,
\end{aligned}
$$

where $Z_{\nu,\mathrm{ss}} = U_\nu N \langle\theta_\nu^2\rangle_0 - \Delta_\nu - i\kappa_\nu$. The expansion for the cavity 2 amplitude α_2 can be directly obtained from Eqs. (S28) - (S30) by interchanging the cavity labels $1 \leftrightarrow 2$. Using these expressions in the mean-field Hamiltonian and keeping only the first-order terms, we find

$$
H_{\mathrm{mf}}[\psi_0 + \delta\psi] = H_{\mathrm{mf}}[\psi_0] + \delta H_{\mathrm{mf}}[\psi_0]\,, \qquad \text{(S31)}
$$

with

$$
\delta H_{\mathrm{mf}}[\psi_0] \approx \hbar \sum_{(\mu,\nu)\in\mathcal{S}} \sum_{(n,m)\in\mathcal{S}} h_{nm,\mu\nu}[\psi_0] \langle\theta_\mu\theta_\nu\rangle_{\delta\psi} \theta_n\theta_m\,. \qquad \text{(S32)}
$$

Here, $\mathcal{S} = \{(1,1),(2,2),(p,1),(p,2),(1,2)\}$ is the set of all possible index tuples and

$$
\begin{aligned}
h_{11,\mu\nu}[\psi_0] &= 2U_1 \mathrm{Re}\left\{a_{1,\mu\nu}\left(\alpha_1[\psi_0]\right)^*\right\}\,, \quad h_{22,\mu\nu}[\psi_0] = 2U_2 \mathrm{Re}\left\{a_{2,\mu\nu}\left(\alpha_2[\psi_0]\right)^*\right\}\,, \\
h_{p1,\mu\nu}[\psi_0] &= 2\sqrt{V_p U_1} \mathrm{Re}\left\{a_{1,\mu\nu}\right\}\,, \quad h_{p2,\mu\nu}[\psi_0] = 2\sqrt{V_p U_2} \mathrm{Re}\left\{a_{2,\mu\nu}\right\}\,, \qquad \text{(S33)} \\
h_{12,\mu\nu}[\psi_0] &= 2\sqrt{U_1 U_2} \mathrm{Re}\left\{\left(a_{1,\mu\nu}\right)^* \alpha_2[\psi_0] + \left(\alpha_1[\psi_0]\right)^* a_{2,\mu\nu}\right\}\,.
\end{aligned}
$$

Plugging (S31) in the equation of motion (S26) and accounting for the steady-state condition (S25), we obtain

$$
i\hbar\frac{\partial}{\partial t}\delta\psi(\boldsymbol{x},t) = \left(-\frac{\hbar^2\nabla^2}{2m} + H_{\mathrm{mf}}[\psi_0]\right)\delta\psi(\boldsymbol{x},t) + \delta H_{\mathrm{mf}}[\psi_0]\psi_0(\boldsymbol{x})\,. \qquad \text{(S34)}
$$

Equation (3) in the main text corresponds to the Fourier transform of this equation. To analyze the spectrum of this dynamical equation, encoding the behaviour of the fluctuation $\delta\psi$, and thus the stability of the fixed point ψ_0, we follow the steps outlined in Ref *(72)*. We first formally integrate (S34), yielding

$$\delta\psi(\boldsymbol{x},t) = e^{-\frac{i}{\hbar}H_0 t}\delta\psi(\boldsymbol{x},t=0) - \frac{i}{\hbar}\int_0^t \mathrm{d}\tau\, e^{-\frac{i}{\hbar}H_0(t-\tau)}\delta H_{\mathrm{mf}}[\psi_0]\psi_0(\boldsymbol{x})\,, \tag{S35}$$

with $H_0 = -(\hbar^2\nabla^2)/(2m) + H_{\mathrm{mf}}[\psi_0]$. Note that the quantity $\delta H_{\mathrm{mf}}[\psi_0]$ depends on the time τ via the variations $\langle\theta_\mu\theta_\nu\rangle_{\delta\psi}$ (see (S32)). Applying the Laplace transform $L[f](s) = \int_0^\infty \mathrm{d}t e^{-st} f(t)$, $s \in \mathbb{C}$, to the solution, while accounting for the convolution theorem of the Laplace transform *(73)*, one obtains

$$\begin{aligned} L[\delta\psi](s) = &\,(s + iH_0/\hbar)^{-1}\,\delta\psi(\boldsymbol{x},t=0) \\ &- i\sum_{(\mu,\nu)\in\mathcal{S}}\sum_{(n,m)\in\mathcal{S}} L[\langle\theta_\mu\theta_\nu\rangle_{\delta\psi}](s) h_{nm,\mu\nu}[\psi_0]\,(s + iH_0/\hbar)^{-1}\,\theta_n\theta_m\psi_0(\boldsymbol{x})\,. \end{aligned} \tag{S36}$$

Together with the Laplace transform of the complex conjugated fluctuation $\delta\psi^*$, we find an equation for the Laplace transformed variation $L[\langle\theta_\alpha\theta_\beta\rangle_{\delta\psi}](s)$:

$$L[\langle\theta_\alpha\theta_\beta\rangle_{\delta\psi}](s) = b_{\alpha\beta}(s) - \sum_{(\mu,\nu)\in\mathcal{S}}\sum_{(n,m)\in\mathcal{S}} C_{\alpha\beta,nm} h_{nm,\mu\nu}[\psi_0] L[\langle\theta_\mu\theta_\nu\rangle_{\delta\psi}](s)\,, \tag{S37}$$

with

$$b_{\alpha\beta}(s) = \int \mathrm{d}\boldsymbol{x}\;\theta_\alpha\theta_\beta\left(\psi_0(s - iH_0/\hbar)^{-1}\delta\psi^*(t=0) + \psi_0^*(s + iH_0/\hbar)^{-1}\delta\psi(t=0)\right)\,, \tag{S38}$$

$$C_{\alpha\beta,nm}(s) = i\int \mathrm{d}\boldsymbol{x}\;\theta_\alpha\theta_\beta\left(\psi_0^*\,(s + iH_0/\hbar)^{-1}\,\theta_n\theta_m\psi_0 - \psi_0\,(s - iH_0/\hbar)^{-1}\,\theta_n\theta_m\psi_0^*\right)\,. \tag{S39}$$

Note that we have omitted here for the sake of presentation the position argument $\boldsymbol{x}$ of the functions appearing in the integrals. Equation (S37) describes a set of five c oupled equations for the Laplace transformed variations $L[\langle\theta_\alpha\theta_\beta\rangle_{\delta\psi}]$, with $(\alpha,\beta)\in\mathcal{S}$. They can be

conveniently combined in a single matrix-vector equation $(1 + C(s)h)\boldsymbol{Y}(s) = \boldsymbol{b}(s)$, where $\boldsymbol{Y}(s) = (L[\langle\hat{\theta}_1^2\rangle_{\delta\psi}](s), L[\langle\hat{\theta}_2^2\rangle_{\delta\psi}](s), L[\langle\hat{\theta}_p\hat{\theta}_1\rangle_{\delta\psi}](s), L[\langle\hat{\theta}_p\hat{\theta}_2\rangle_{\delta\psi}](s), L[\langle\hat{\theta}_1\hat{\theta}_2\rangle_{\delta\psi}](s))^T$ and the entries of the vector $\boldsymbol{b}$, and the matrices C and h are given in Eqs. (S33), (S38), and (S39). The stability of the fixed point can be determined by analyzing the solutions s of the dispersion relation

$$\det(1 + C(s)h) = 0\,, \tag{S40}$$

as discussed in Ref *(72)* and in the following section.

Extracting the subradiant state's lifetime

The subradiant state represents a fixed point of the GGPE (S20), thus fulfilling the steady-state condition (S25). As such, we can employ the linear stability analysis of the preceding section to determine its stability for given parameters. As we will discuss in the following, this allows us to predict its lifetime in the presence of atom losses.

In Fig. 4 of the main text, we study quenches from cavity 2 to 1. Here, the subradiant state of the system (after performing the quench) is characterized by a spatial configuration that supports the scattering of photons into cavity 2 but suppresses scattering into cavity 1. The corresponding steady state ψ_0 can be computed by solving the steady-state condition (S25) for the cavity detunings after the quench, $(\Delta_1, \Delta_2) = (0, \delta)$, and for vanishing coupling to cavity 1, $g_1 = 0$. We verify that this state obeys our definition of the subradiant state, that is, $\langle\theta_p\theta_1\rangle_0 = \langle\theta_1\theta_2\rangle_0 = 0$. Hence, the spatial configuration prohibits the occupation of this cavity, such that $\alpha_1[\psi_0] = 0$, and the state ψ_0 remains a steady state of the dynamical equation, even in the presence of the coupling to cavity 1, $g_1 \neq 0$. By numerically computing the roots of the dispersion relation (S40) for the state ψ_0 and for $g_1 \neq 0$, we determine its stability. A root with positive real part $\gamma > 0$ indicates that the coupling to cavity 1 renders the subradiant state unstable, otherwise it remains stable. Repeating this calculation for different quench strengths

δ and atom numbers N, we construct the stability diagram reported in Fig. 4**F** in the main text.

As visible from this stability diagram, we can identify for each considered quench strength δ a critical atom number $N_c(\delta)$ below which the subradiant state becomes unstable ($\gamma > 0$). Suppose that the atom number after the quench is N_0 and takes values between 2.43×10^5 and 2.97×10^5, corresponding to the typical range of atom numbers in the experiment. Then the subradiant state represents a stable configuration if $N_0 > N_c$. Even if initially fulfilled, atom losses eventually decrease the atom number over time, $N(t) \leq N_0$, and the subradiant state becomes unstable when $N(t^*) = N_c$. Thus, the atom loss brings the system from the stable region ($\gamma \leq 0$) to the unstable region ($\gamma > 0$) in the stability diagram, as indicated by the green arrow in Fig. 4**F**. The delay times shown in Fig. 4**E** represent essentially the length of the time interval it takes the atom losses to decrease the initial atom number below the critical value. Imposing as above an exponential decay of the atom number, $N(t) = N_0 e^{-\Gamma t}$, we can predict the delay time t^* by solving the condition $N_0 e^{-\Gamma t^*} = N_c$, yielding

$$t^*(\delta) = -\frac{1}{\Gamma} \ln \left(\frac{N_c(\delta)}{N_0} \right) . \tag{S41}$$

It is important to note that this equation only allows us to make viable predictions about the delay time if the dynamical system state $\psi(t)$ remains close to the predicted steady subradiant state ψ_0 after the quench. In particular, as the latter is a function of the atom number, $\psi_0 = \psi_0(N)$, the subradiant state evolves over time when atom loss is present. As the atom loss occurs here on a much longer timescale than the one associated with the long-range interaction, the system - if initially trapped in the subradiant state - is able to follow adiabatically the evolving subradiant state.

Experimental Details

Atomic loss rate

We measure the loss rate of the system by preparing the cloud in the crossed beam dipole trap and ramping up the transverse pump lattice. We let the system evolve for a certain hold time before we switch off all potentials and measure the remaining atom number after ballistic expansion. Typical decay curves are shown in Figure S2 for different parameters.

Measurement protocol of the subradiant states

In the section "Formation of Subradiant States" in the main text, we present a measurement protocol which extracts a hysteresis region of subradiant states. For certain cavity 1 and 2 detunings, we linearly ramp up the transverse pump power in $5\,\mathrm{ms}$ such that the atoms self-organize into the initial cavity. Next, over $25\,\mathrm{ms}$, the cavity detunings are swept across the phase boundary. The sweep is symmetric with regards to the phase boundary: one of the ramps linearly changes $\Delta_{1,2}$ from $[0.5, -0.5]\,2\pi \times \mathrm{MHz}$ to $[-0.5, 0.5]\,2\pi \times \mathrm{MHz}$ and yields $\Delta_1 - \Delta_2$ as a unique parameter characterizing this sweep. At the sweep half-time of $12.5\,\mathrm{ms}$, the cavity detunings are degenerate $\Delta_1 - \Delta_2 = 0$. We extract the switching time from traces as shown in Fig. 3**A**, which corresponds to a unique value of $\Delta_1 - \Delta_2$. We repeat this measurement for different values of $(\Delta_1 + \Delta_2)/2$ and show the results in Fig. 3**B**.

In the section "Lifetime of Subradiant States" in the main text, we characterize the lifetimes of the subradiant system by quenching across the phase boundary between the two cavities and determining the time after which the system switches between the two cavities (see Fig. 4). At the start of each quench measurement, a $10\,\mathrm{ms}$ long transverse pump ramp is used to prepare a self-organized state in one of the cavities. The detunings of the cavities determine which cavity gets populated. When quenching from cavity 2 to 1, cavity 2 is initially resonant with respect to the transverse pump, so $\Delta_2 = 0$ and cavity 1 is detuned to $\Delta_1 = [-0.6, -0.1]\,2\pi \times \mathrm{MHz}$. After

the transverse pump ramp, we hold the final pumping strength and cavity 1 is quenched onto resonance $\Delta_1 = 0$ and cavity 2 away from resonance by $\Delta_2 = [-0.6, -0.1]\, 2\pi \times \mathrm{MHz}$. Typical photon traces for both cavities are shown in Fig. 4**A, C**. Following a buildup of the photon level in cavity 2, the system stays self-organized in cavity 2 beyond the quench. It remains subradiant until a characteristic delay time, when the self-organization jumps from the second to the first cavity. We extract this delay time from numerically fitting a delta function in cavity 2 to the decay and in cavity 1 to the onset of the photon field. We find that both times lay within $0.1\,\mathrm{ms}$. We nevertheless take the average of both as the delay time plotted in Fig. 4**E**.

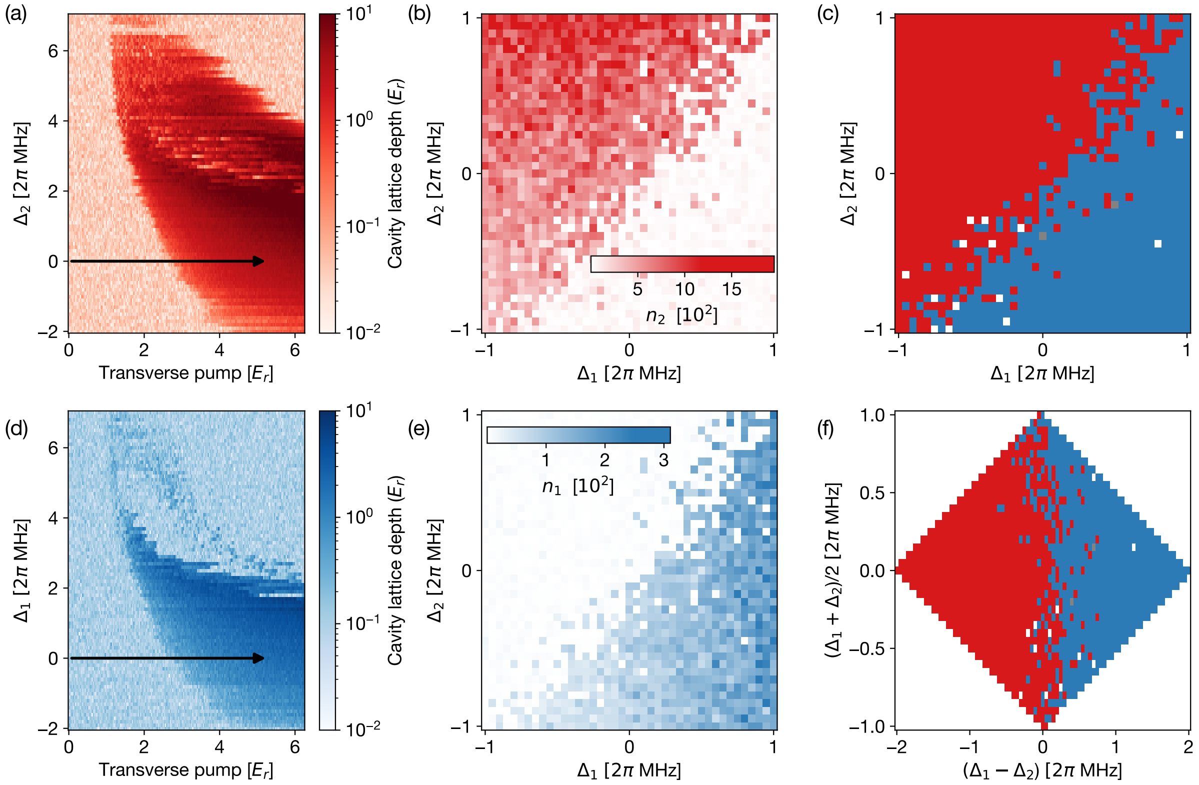

Figure S1: **Measuring Phase Diagrams. (a,d)** Single cavity phase diagrams for cavity 2 (1). We linearly ramp up the transverse pump to 6.2 E_r and record the cavity lattice depth for different detunings Δ_2 (Δ_1). For these measurements, cavity 1 (2) is detuned far away from resonance and does not influence the self-organisation of cavity 2 (1). Black arrows highlight transverse pump ramps, where we stop the ramp at 5.1 E_r and collect data for the subsequent panels. **(b,e)** Measured intracavity photon number $n_{1(2)}$ after increasing $\hbar V_p$ linearly from 0 to 5.1 E_r at fixed detunings $\Delta_{1(2)}$ **(c)** Combined and binarized phase diagram derived from panels **(b,e)** by defining thresholds of $(n_1^{\text{th}}, n_2^{\text{th}}) = (30, 200)$ photons for identifying superradiance in a respective cavity. The steady-state photon numbers, and thus defined threshold values, differ due to the different cavity decay rates. Blue (red) pixels indicate superradiance in cavity 1 (2), white pixels indicate both cavity populations below threshold, grey pixels indicate coexistence where both cavity populations are above the respective threshold. **(f)** Same data as in panel **(c)**, but in rotated coordinates of relative cavity detunings $\Delta_1 - \Delta_2$ and mean cavity detunings $(\Delta_1 + \Delta_2)/2$ as used in the main text. White spaces outside the square indicate no data.

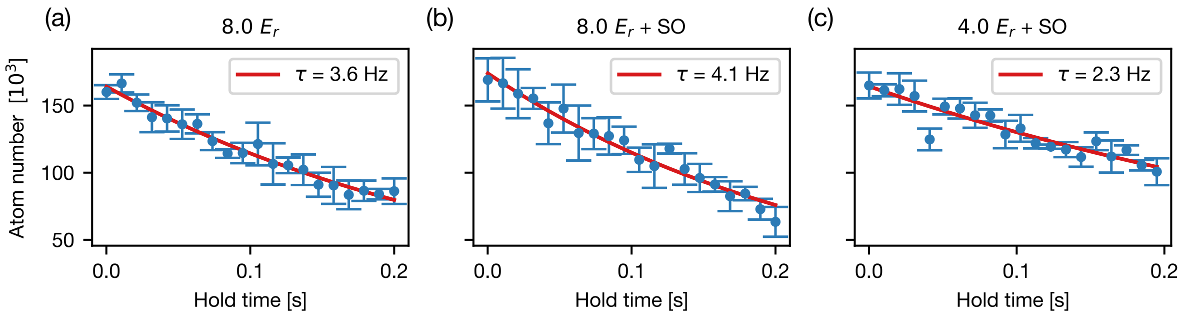

Figure S2: **Atom loss.** The transverse pump drive is linearly ramped up in 10 ms from 0.0 to 8.0 E_r for **(a,b)** and from 0 to 4.0 E_r for **(c)**. After variable hold time, the atom numbers are extracted from time-of-flight images of the cloud. The cavity 1 detuning in **(a)** is detuned away from resonance and atoms do not self-organise. For **(b,c)**, the cavity 1 detuning is set to resonance such that atoms self-order during the transverse pump ramp. In all measurements cavity 2 is far detuned from resonance. This figure estimates atom loss rates of up to 4 Hz present in our experiment, which have been taken into account in the numerical models.

REFERENCES AND NOTES

1. R. H. Dicke, Coherence in spontaneous radiation processes. *Phys. Rev.* **93**, 99–110 (1954).

2. M. Gross, S. Haroche, Superradiance: An essay on the theory of collective spontaneous emission. *Phys. Rep.* **93**, 301–396 (1982).

3. T. Brandes, Coherent and collective quantum optical effects in mesoscopic systems. *Phys. Rep.* **408**, 315–474 (2005).

4. M. Reitz, C. Sommer, C. Genes, Cooperative quantum phenomena in light-matter platforms. *PRX Quantum* **3**, 010201 (2022).

5. A. Crubellier, S. Liberman, D. Pavolini, P. Pillet, Superradiance and subradiance: I. interatomic interference and symmetry properties in three-level systems. *J. Phys. B At. Mol. Phys* **18**, 3811–3833 (1985).

6. R. G. DeVoe, R. G. Brewer, Observation of superradiant and subradiant spontaneous emission of two trapped ions. *Phys. Rev. Lett.* **76**, 2049–2052 (1996).

7. M. D. Barnes, P. S. Krstic, P. Kumar, A. Mehta, J. C. Wells, Far-field modulation of fluorescence decay rates in pairs of oriented semiconducting polymer nanostructures. *Phys. Rev. B* **71**, 241303 (2005).

8. S. Filipp, A. F. van Loo, M. Baur, L. Steffen, A. Wallraff, Preparation of subradiant states using local qubit control in circuit qed. *Phys. Rev. A* **84**, 061805 (2011).

9. B. H. McGuyer, M. McDonald, G. Z. Iwata, M. G. Tarallo, W. Skomorowski, R. Moszynski, T. Zelevinsky, Precise study of asymptotic physics with subradiant ultracold molecules. *Nat. Phys.* **11**, 32–36 (2015).

10. R. Reimann, W. Alt, T. Kampschulte, T. Macha, L. Ratschbacher, N. Thau, S. Yoon, D. Meschede, Cavity-modified collective Rayleigh scattering of two atoms. *Phys. Rev. Lett.* **114**, 023601 (2015).

11. M. Verde, A. Schaefer, B. Zenz, Z. Shehata, S. Richter, C. T. Schmiegelow, J. von Zanthier, F. Schmidt-Kaler, Spin-selective coherent light scattering from ion crystals. arXiv:2404.12513 [physics.atom-ph] (2024).

12. Y. Sonnefraud, N. Verellen, H. Sobhani, G. A. Vandenbosch, V. V. Moshchalkov, P. Van Dorpe, P. Nordlander, S. A. Maier, Experimental realization of subradiant, superradiant, and fano resonances in ring/disk plasmonic nanocavities. *ACS Nano* **4**, 1664–1670 (2010).

13. G. Ferioli, A. Glicenstein, I. Ferrier-Barbut, A. Browaeys, A non-equilibrium superradiant phase transition in free space. *Nat. Phys.* **19**, 1345–1349 (2023).

14. S. Fernández-Vidal, S. Zippilli, G. Morigi, Nonlinear optics with two trapped atoms. *Phys. Rev. A* **76**, 053829 (2007).

15. A. Neuzner, M. Körber, O. Morin, S. Ritter, G. Rempe, Interference and dynamics of light from a distance-controlled atom pair in an optical cavity. *Nat. Photon.* **10**, 303–306 (2016).

16. S. Inouye, A. P. Chikkatur, D. M. Stamper-Kurn, J. Stenger, D. E. Pritchard, W. Ketterle, Superradiant rayleigh scattering from a Bose-Einstein condensate. *Science* **285**, 571–574 (1999).

17. H. Ritsch, P. Domokos, F. Brennecke, T. Esslinger, Cold atoms in cavity-generated dynamical optical potentials. *Rev. Mod. Phys.* **85**, 553–601 (2013).

18. F. Mivehvar, F. Piazza, T. Donner, H. Ritsch, Cavity qed with quantum gases: New paradigms in many-body physics. *Adv. Phys.* **70**, 1–153 (2021).

19. K. Hepp, E. Lieb, On the superradiant phase transition for molecules in a quantized radiation field: The Dicke maser model. *Ann. Phys. Rehabil. Med.* **76**, 360–404 (1973).

20. Y. K. Wang, F. T. Hioe, Phase transition in the Dicke model of superradiance. *Phys. Rev. A* **7**, 831–836 (1973).

21. K. Baumann, C. Guerlin, F. Brennecke, T. Esslinger, Dicke quantum phase transition with a superfluid gas in an optical cavity. *Nature* **464**, 1301–1306 (2010).

22. B. M. Garraway, The Dicke model in quantum optics: Dicke model revisited. *Philos. Trans. R. Soc. A Math. Phys. Eng. Sci.* **369**, 1137–1155 (2011).

23. P. Nataf, C. Ciuti, No-go theorem for superradiant quantum phase transitions in cavity qed and counter-example in circuit qed. *Nat. Commun.* **1**, 72 (2010).

24. W. Guerin, M. O. Araújo, R. Kaiser, Subradiance in a large cloud of cold atoms. *Phys. Rev. Lett.* **116**, 083601 (2016).

25. A. Cipris, N. A. Moreira, T. S. do Espirito Santo, P. Weiss, C. J. Villas-Boas, R. Kaiser, W. Guerin, R. Bachelard, Subradiance with saturated atoms: Population enhancement of the long-lived states. *Phys. Rev. Lett.* **126**, 103604 (2021).

26. G. Ferioli, A. Glicenstein, L. Henriet, I. Ferrier-Barbut, A. Browaeys, Storage and release of subradiant excitations in a dense atomic cloud. *Phys. Rev. X* **11**, 021031 (2021).

27. A. Glicenstein, G. Ferioli, A. Browaeys, I. Ferrier-Barbut, From superradiance to subradiance: Exploring the many-body Dicke ladder. *Opt. Lett.* **47**, 1541–1544 (2022).

28. M. M. Cola, D. Bigerni, N. Piovella, Recoil-induced subradiance in an ultracold atomic gas. *Phys. Rev. A* **79**, 053622 (2009).

29. P. Wolf, S. C. Schuster, D. Schmidt, S. Slama, C. Zimmermann, Observation of subradiant atomic momentum states with Bose-Einstein condensates in a recoil resolving optical ring resonator. *Phys. Rev. Lett.* **121**, 173602 (2018).

30. Z. Yan, J. Ho, Y.-H. Lu, S. J. Masson, A. Asenjo-Garcia, D. M. Stamper-Kurn, Superradiant and subradiant cavity scattering by atom arrays. *Phys. Rev. Lett.* **131**, 253603 (2023).

31. J. Rui, D. Wei, A. Rubio-Abadal, S. Hollerith, J. Zeiher, D. M. Stamper-Kurn, C. Gross, I. Bloch, A subradiant optical mirror formed by a single structured atomic layer. *Nature* **583**, 369–374 (2020).

32. A. Campa, T. Dauxois, S. Ruffo, Statistical mechanics and dynamics of solvable models with long-range interactions. *Phys. Rep.* **480**, 57–159 (2009).

33. N. Defenu, T. Donner, T. Macrí, G. Pagano, S. Ruffo, A. Trombettoni, Long-range interacting quantum systems. *Rev. Mod. Phys.* **95**, 035002 (2023).

34. N. Defenu, A. Lerose, S. Pappalardi, Out-of-equilibrium dynamics of quantum many-body systems with long-range interactions. *Phys. Rep.* **1074**, 1–92 (2024).

35. K. Jain, F. Bouchet, D. Mukamel, Relaxation times of unstable states in systems with long range interactions. *J. Stat. Mech.* **2007**, P11008 (2007).

36. A. Campa, P.-H. Chavanis, A. Giansanti, G. Morelli, Dynamical phase transitions in long-range hamiltonian systems and tsallis distributions with a time-dependent index. *Phys. Rev. E* **78**, 040102 (2008).

37. D. Lynden-Bell, R. Wood, A. Royal, The gravo-thermal catastrophe in isothermal spheres and the onset of red-giant structure for stellar systems. *Mont. Not. R. Astronom. Soc.* **138**, 495–525 (1968).

38. J. Barré, T. Dauxois, G. De Ninno, D. Fanelli, S. Ruffo, Statistical theory of high-gain free-electron laser saturation. *Phys. Rev. E* **69**, 045501 (2004).

39. A. Morales, P. Zupancic, J. Léonard, T. Esslinger, T. Donner, Coupling two order parameters in a quantum gas. *Nat. Mater.* **17**, 686–690 (2018).

40. P. Zupancic, D. Dreon, X. Li, A. Baumgärtner, A. Morales, W. Zheng, N. R. Cooper, T. Esslinger, T. Donner, P-band induced self-organization and dynamics with repulsively driven ultracold atoms in an optical cavity. *Phys. Rev. Lett.* **123**, 233601 (2019).

41. S. Zippilli, G. Morigi, H. Ritsch, Suppression of bragg scattering by collective interference of spatially ordered atoms with a high-q cavity mode. *Phys. Rev. Lett.* **93**, 123002 (2004).

42. M. Gegg, A. Carmele, A. Knorr, M. Richter, Superradiant to subradiant phase transition in the open system Dicke model: Dark state cascades. *New J. Phys.* **20**, 013006 (2018).

43. A. Shankar, J. T. Reilly, S. B. Jäger, M. J. Holland, Subradiant-to-subradiant phase transition in the bad cavity laser. *Phys. Rev. Lett.* **127**, 073603 (2021).

44. C. Hotter, L. Ostermann, H. Ritsch, Cavity sub- and superradiance for transversely driven atomic ensembles. *Phys. Rev. Res.* **5**, 013056 (2023).

45. R. Plestid, D. H. J. O'Dell, Balancing long-range interactions and quantum pressure: Solitons in the hamiltonian mean-field model. *Phys. Rev. E* **100**, 022216 (2019).

46. R. Balescu, Irreversible processes in ionized gases. *Phys. Fluids* **3**, 52–63 (1960).

47. M. Henon, Vlasov equation. *Astron. Astrophys.* **114**, 211 (1982).

48. P. Bertrand, D. Sarto, A. Ghizzo, *The Vlasov Equation 1: History and General Properties* (Wiley, 2019).

49. Y. Elskens, M. K. H. Kiessling, Microscopic foundations of kinetic plasma theory: The relativistic vlasov–maxwell equations and their radiation-reaction-corrected generalization. *J. Stat. Phys.* **180**, 749–772 (2020).

50. D. Lynden-Bell, Statistical mechanics of violent relaxation in stellar systems. *Mont. Not. R. Astronom. Soc.* **136**, 101–121 (1967).

51. P. H. Chavanis, On the lifetime of metastable states in self-gravitating systems. *Astronom. Astrophys.* **432**, 117–138 (2005).

52. E. Saadat, I. Latella, S. Ruffo, Lifetime of locally stable states near a phase transition in the thirring model. *J. Stat. Mech.* **2023**, 083207 (2023).

53. T. N. Teles, F. P. da C. Benetti, R. Pakter, Y. Levin, Nonequilibrium phase transitions in systems with long-range interactions. *Phys. Rev. Lett.* **109**, 230601 (2012).

54. P. H. Chavanis, Phase transitions in self-gravitating systems. *Int. J. Mod. Phys. B* **20**, 3113–3198 (2006).

55. S. Gupta, D. Mukamel, Slow relaxation in long-range interacting systems with stochastic dynamics. *Phys. Rev. Lett.* **105**, 040602 (2010).

56. M. C. Braidotti, M. Lovisetto, R. Prizia, C. Michel, C. Didier, M. Bellec, E. M. Wright, B. Marcos, D. Faccio, Experimental observation of violent relaxation. *Commun. Phys.* **7**, 206 (2024).

57. A. Pikovsky, S. Gupta, T. N. Teles, F. P. C. Benetti, R. Pakter, Y. Levin, S. Ruffo, Ensemble inequivalence in a mean-field *xy* model with ferromagnetic and nematic couplings. *Phys. Rev. E* **90**, 062141 (2014).

58. M. F. P. Silva, T. M. R. Filho, Y. Elskens, Critical exponent for the lyapunov exponent and phase transitions in the generalized hamiltonian mean-field model. *J. Phys. A Math Theor.* **53**, 215001 (2020).

59. T. Keller, V. Torggler, S. B. Jäger, S. Schütz, H. Ritsch, G. Morigi, Quenches across the self-organization transition in multimode cavities. *New J. Phys.* **20**, 025004 (2018).

60. Z. Wu, J. Fan, X. Zhang, J. Qi, H. Wu, Signatures of prethermalization in a quenched cavity-mediated long-range interacting fermi gas. *Phys. Rev. Lett.* **131**, 243401 (2023).

61. T. Zwettler, G. del Pace, F. Marijanovic, S. Chattopadhyay, T. Bühler, C.-M. Halati, L. Skolc, L. Tolle, V. Helson, G. Bolognini, A. Fabre, S. Uchino, T. Giamarchi, E. Demler, J. P. Brantut, Nonequilibrium dynamics of long-range interacting fermions. *Phys. Rev. X* **15**, 021089 (2025).

62. F. Marijanović, S. Chattopadhyay, L. Skolc, T. Zwettler, C.-M. Halati, S. B. Jäger, T. Giamarchi, J.-P. Brantut, E. Demler, Dynamical instabilities of strongly interacting ultracold fermions in an optical cavity. arXiv:2406.13548 [cond-mat.quant-gas] (2024).

63. S. Schütz, G. Morigi, Prethermalization of atoms due to photon-mediated long-range interactions. *Phys. Rev. Lett.* **113**, 203002 (2014).

64. A. Morales, D. Dreon, X. Li, A. Baumgärtner, P. Zupancic, T. Donner, T. Esslinger, Two-mode Dicke model from nondegenerate polarization modes. *Phys. Rev. A* **100**, 013816 (2019).

65. X. Li, D. Dreon, P. Zupancic, A. Baumgärtner, A. Morales, W. Zheng, N. R. Cooper, T. Donner, T. Esslinger, First order phase transition between two centro-symmetric superradiant crystals. *Phys. Rev. Res.* **3**, L012024 (2021).

66. S. Schütz, H. Habibian, G. Morigi, Cooling of atomic ensembles in optical cavities: Semiclassical limit. *Phys. Rev. A* **88**, 033427 (2013).

67. S. B. Jäger, T. Schmit, G. Morigi, M. J. Holland, R. Betzholz, Lindblad master equations for quantum systems coupled to dissipative bosonic modes. *Phys. Rev. Lett.* **129**, 063601 (2022).

68. J. Larson, S. Fernández-Vidal, G. Morigi, M. Lewenstein, Quantum stability of mott-insulator states of ultracold atoms in optical resonators. *New J. Phys.* **10**, 045002 (2008).

69. S. Krämer, D. Plankensteiner, L. Ostermann, H. Ritsch, Quantumoptics.jl: A julia framework for simulating open quantum systems. *Comput. Phys. Commun.* **227**, 109–116 (2018).

70. L. Pitaevskii, S. Stringari, *Bose-Einstein Condensation* (Oxford Univ. Press, 2003).

71. C. Gros, *Complex and Adaptive Dynamical Systems: A Primer* (Springer International Publishing, 2015).

72. S. B. Jäger, J. Cooper, M. J. Holland, G. Morigi, Dynamical phase transitions to optomechanical superradiance. *Phys. Rev. Lett.* **123**, 053601 (2019).

73. J. L. Schiff, *The Laplace Transform* (Springer, 1999).
